# In Operando Locally‐Resolved Photophysics in Perovskite Solar Cells by Correlation Clustering Imaging

**DOI:** 10.1002/adma.202413126

**Published:** 2024-12-29

**Authors:** Boris Louis, Sudipta Seth, Qingzhi An, Ran Ji, Yana Vaynzof, Johan Hofkens, Ivan G. Scheblykin

**Affiliations:** ^1^ Division of Chemical Physics and NanoLund Lund University PO Box 124 Lund 22100 Sweden; ^2^ Laboratory for Photochemistry and Spectroscopy Division for Molecular Imaging and Photonics Department of Chemistry Katholieke Universiteit Leuven Leuven 3001 Belgium; ^3^ Chair for Emerging Electronic Technologies Technical University of Dresden Nöthnitzer Str. 61 01187 Dresden Germany; ^4^ Leibniz‐Institute for Solid State and Materials Research Dresden Helmholtzstraße 20 01069 Dresden Germany; ^5^ Max Planck Institute for Polymer Research 55128 Mainz Germany

**Keywords:** correlative microscopy, functional imaging, Inoperando solar cell investigation, microspectroscopy, optical fluctuation

## Abstract

The instability of metal halide perovskites limits the commercialization of solar cells despite their impressive efficiencies. This instability, driven by photo‐induced ion migration, leads to material restructuring, defect formation, degradation, and defect healing. However, these same “unwanted” properties enable to propose Correlation Clustering Imaging (CLIM), a technique that detects local photoluminescence (PL) fluctuations through wide‐field fluorescence microscopy. It is shown that such fluctuations are present in high‐quality perovskites and their corresponding solar cells. CLIM successfully visualizes the polycrystalline grain structure in perovskite films, closely matching electron microscopy images. The analysis of fluctuations reveals a dominant metastable defect responsible for the fluctuations. In solar cells in short‐circuit conditions, these fluctuations are significantly larger, and corresponding correlated regions extend up to 10 micrometers, compared to 2 micrometers in films. It is proposed that the regions resolved by CLIM in solar cells possess a common pool of charge extraction channels, which fluctuate and cause PL to vary. Since PL fluctuations reflect non‐radiative recombination processes, CLIM provides valuable insights into the structural and functional dynamics of carrier transport, ion migration, defect behavior, and recombination losses. CLIM offers a non‐invasive approach to understanding luminescent materials and devices in operando, utilizing contrasts based on previously untapped properties.

## Graphical abstract

Correlation clustering imaging (CLIM) exploits local intensity fluctuations observed by fluorescence microscopy in metal‐halide perovskite films and their solar cells to generate novel contrasts uncovering the spatial dynamics of charge carrier transport, recombination losses, and material restructuring.

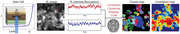

## 1 Introduction

The challenge of correlating materials’ structure to their functional properties is a cornerstone issue in material sciences. Unlocking this structure‐function relationship is essential for rational material and device design, leading to improved properties and efficiencies.^[^ ^1^, ^2^, ^3^, ^4^, ^5^, ^6^ ^]^ Existing characterization methods often focus singularly on the materials' structure or physical properties. Therefore, comprehensive understanding necessitates applying multiple methods to a single sample, which complicates the characterization process and introduces unavoidable artifacts. Techniques like scanning electron microscopy (SEM), transmission electron microscopy (TEM), and synchrotron‐based methods often alter material properties due to their operational conditions.^[^ ^7^, ^8^, ^9^, ^10^ ^]^ In contrast, light‐based methods such as absorption, photoluminescence (PL) microscopy, and spectroscopy are much less invasive but suffer from limited resolution.^[^ ^11^, ^12^, ^13^, ^14^ ^]^

Super‐resolution microscopy techniques based on fluorescence labeling by dyes and nanoparticles are very important for biological sciences^[^ ^15^, ^16^, ^17^, ^18^ ^]^ but have less impact on material science. This is because applying fluorescence labeling to materials and devices is often challenging or simply impossible. Moreover, labeling is invasive because it can easily alter the electronic properties of the material under study. At the same time, using intrinsic luminescence of materials is often problematic due to the extremely high density and spatial mobility of the emitting species. Consequently, SEM combined with PL microscopy remains the common tool for structural and functional characterization of luminescent materials,^[^ ^19^, ^20^, ^21^, ^22^, ^23^, ^24^, ^25^ ^]^ which, however, are not suitable when the materials are integrated into multilayered optoelectronic devices like solar cells. Analyzing these devices during operation necessitates non‐invasive techniques that can penetrate through layers and simultaneously assess morphology and optoelectronic properties.

Many super‐resolution imaging methods rely on dye labels whose fluorescence switches on and off stochastically – the phenomenon known as fluorescence blinking. While for biological imaging, the blinking phenomenon is just a tool to improve spatial resolution, in materials, the situation is different. Indeed, PL blinking/fluctuations provide insights into the nature of the excited states, their diffusion, photochemical process, and material dynamics,^[^ ^26^, ^27^, ^28^, ^29^, ^30^ ^]^ which are vital information for fundamental and applied materials science and engineering. Various material systems exhibit PL fluctuations when their particle size decreases below 1 micrometer, such as individual conjugated polymer molecules and their aggregates,^[^ ^31^ ^]^ aggregates of organic dyes,^[^ ^32^ ^]^ and individual semiconductor nano and microcrystals.^[^ ^26^, ^27^, ^28^, ^33^, ^34^, ^35^, ^36^ ^]^ The diversity of blinking systems shows that this phenomenon is by far not exclusive to single quantum systems and single emitters.^[^ ^37^ ^]^ Analyzing these fluctuations alongside imaging (including super‐resolution) and spectroscopic measurements offers critical information about the photophysics of these materials at the nanoscale.^[^ ^26^, ^27^, ^28^, ^29^, ^30^ ^]^ Unfortunately, direct application of the same ideas to solid films and other bulk samples is ineffective as the local PL fluctuations become quite small or absent due to signal overlap from many structural units caused by the poor spatial resolution of optical microscopy (the ensemble averaging effect). Therefore, alternative approaches utilizing these effects for functional imaging are needed.

In fact, several important functional material classes exhibit detectable temporal variations of local PL resolved by optical microscopy, even in their thin films and other large structures. These include 2D materials^[^ ^38^, ^39^, ^40^ ^]^ and various metal halide perovskites (MHPs),^[^ ^27^, ^33^, ^34^, ^35^, ^41^, ^42^ ^]^ which have numerous applications.^[^ ^43^, ^44^, ^45^ ^]^ The intrinsic feature of MHPs is the presence of nano/microscale heterogeneity stemming from grains and grain boundaries in both perovskite films and devices, impacting their performance and stability, underscoring the critical need to connect their structure and function.^[^ ^12^, ^20^, ^46^, ^47^, ^48^ ^]^ Consequently, high‐resolution functional imaging methods are in great demand for fundamental and applied studies of contemporary electronic materials and devices based on them.

To address this demand, we developed the Correlation Clustering Imaging (CLIM) method, which analyzes the temporal evolution of fluorescence microscopy time‐dependent images (movie) acquired by a wide‐field fluorescence microscope. CLIM clusters pixels in the PL image based on the time correlation in their PL intensity variations. This method extracts structural information and optoelectronic properties from the kinetics of fluctuations in a single, non‐invasive measurement. Thus, CLIM gives image contrasts based on properties that have never been used for this purpose. We demonstrate CLIM on methylammonium lead triiodide (MAPbI_3_) perovskite thin films and solar cells under operation. CLIM images and subsequent analysis of the PL properties of the revealed local clusters shed light on the nature of nonradiative recombination in the perovskite and the local dynamics of charge carrier extraction in the photovoltaic devices.

## 2 Results

### 2.1 Functional Imaging Method: Correlation Clustering Imaging (CLIM)

The Correlation Clustering Imaging (CLIM) algorithm uses spatiotemporal intensity fluctuations in wide‐field fluorescence microscopy movies of thin films made from metastable fluorescent materials. The optical setup used for the measurement is depicted in Note SI (Supporting Information). By analyzing temporal correlations between neighboring pixels, CLIM segments the image into regions (clusters of pixels) that exhibit synchronous fluctuations. As a result of further analysis, the whole movie (a set of time‐dependent PL images) is converted to several static images with contrasts carrying information about the local temporal dynamics of the sample.

**Figure 1:**
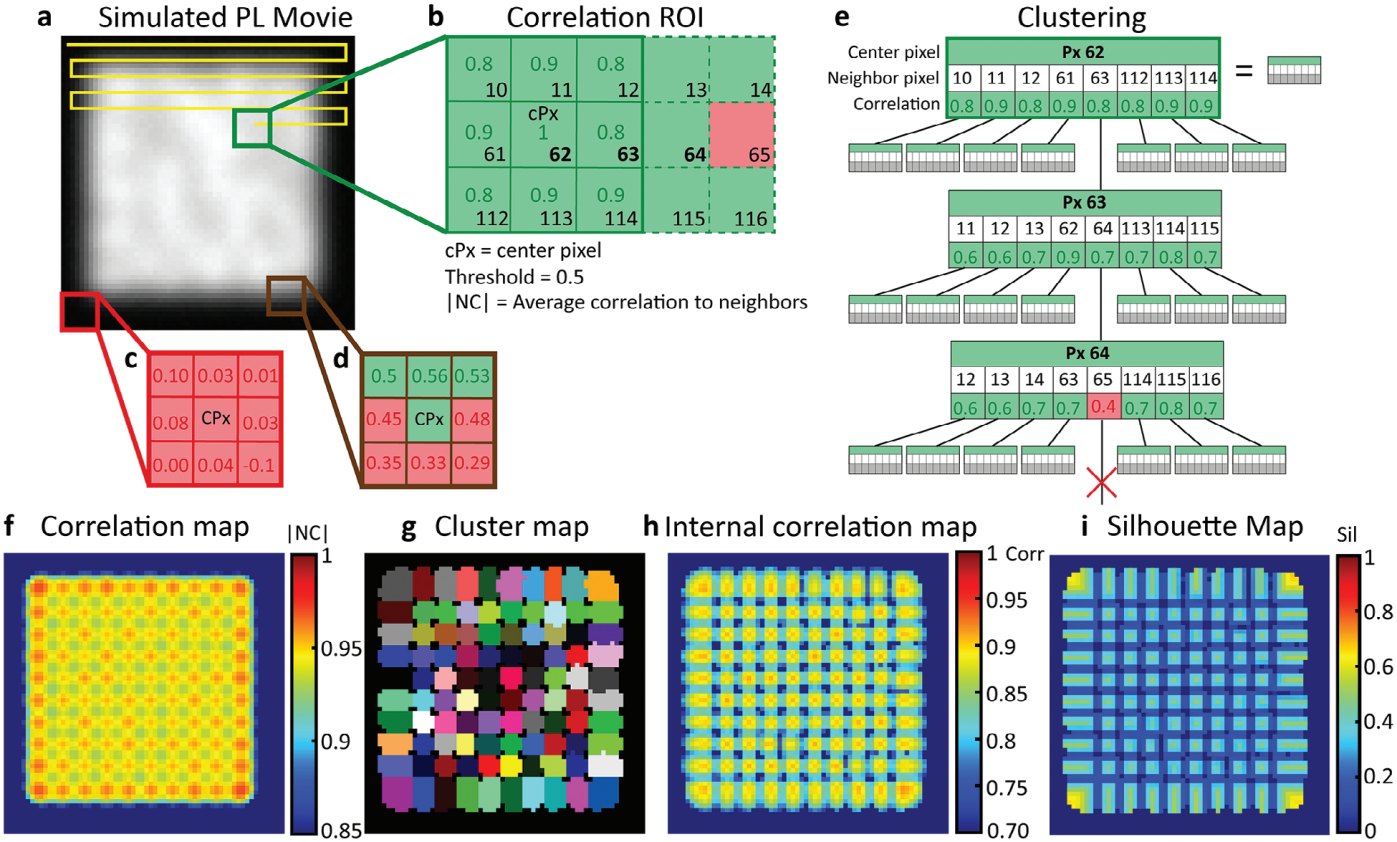
CLIM method. a) Simulated fluorescence image featuring an array of blinking emitters, mimicking a polycrystalline perovskite film. b–d) Illustration of 3x3 ROI (region of interest) used for calculating the correlations of the central pixel (CPx) with its neighboring pixels (px). Values in green/red indicate that the correlation was above/below the chosen correlation threshold (0.5), respectively. e) Clustering procedure: The pixel with the highest correlation to its neighbors becomes the seeding pixel (e.g. px 62) for the growth of a new cluster. The cluster is grown by considering the correlation of the seeding pixel (e.g. px 62) with the neighbors (e.g. px 63, 112 … and others), and then their neighbors like a “family tree”. The cluster is grown as long as pixels with the correlation above threshold (e.g. 0.5) are found. When none can be found, the current cluster is complete, and a new cluster is initiated starting from a new seeding pixel. In the given example it happens at px 64 where the correlation with pixel 65 is below the threshold leading to the stopping of the process for that branch of the “tree”. f) Correlation map: it displays the average correlation of each pixel with their neighbors. g) Cluster map: the result of the clustering in which each color represents a highly correlated region. Note that some clusters share the same color due to limitations in the color pallet. h) Internal correlation map: it displays the average correlation of the pixels to their assigned cluster and provides the “quality” of the clustering. i) Silhouette map: it indicates the extent to which a pixel is more correlated with the cluster it is assigned to in comparison with its correlation to other neighboring clusters.

**Figure** **1** illustrates this method using a simulated movie (Movie S1, Supporting Information) featuring an array of independently blinking emitters. These emitters are positioned in proximity to simulate a luminescent film with distinct grains emitting independently (Figure 1a). The grain size is comparable to the microscope's point spread function (PSF). Simulation details are provided in Note SII (Supporting Information). As shown in Figure 1a, the individual emitters cannot be distinguished in the simulated PL image due to the limited spatial resolution.

The algorithm is described in Note SIII (Supporting Information). Briefly, the movie is scanned over the image using a region of interest (ROI) consisting of 3×3 pixels, as shown in Figure 1a,b. For each ROI, the Pearson correlation coefficients^[^ ^49^ ^]^ in time between the ROI's central pixel (cPx) and its direct neighboring pixels are computed, yielding 8 correlation values per pixel. Figure 1b, c provides examples of such ROI for the movie's high and low correlation regions. The pixel with the highest average correlation to its neighbor is chosen as the seeding pixel for the growth of a new cluster (e.g., pixel 62 in Figure 1e). The correlation values between the seeding pixel and its neighbors are compared to the correlation threshold (automatically determined by the algorithm, see Note SIII, Supporting Information), and the neighboring pixels are added to the cluster currently in growth, if their correlation value is higher than the said threshold. Then, the neighbors of the newly added pixels to the cluster are inspected to determine if their correlations are also higher than the correlation threshold. To ensure cluster consistency, the correlation between the newly added pixels and those already assigned to the cluster in growth is also determined and compared to the threshold. The search is extended from neighbor to neighbor, akin to reconstructing a family tree (see Figure 1e), until no pixel satisfying both the correlation threshold condition, and the cluster consistency condition can be found. In this case, a new cluster is created starting from the pixel with the highest average correlation to its neighbors amongst the pixels that have not yet been assigned to a cluster. The procedure continues until all pixels are assigned to a cluster (a region of high temporal correlation in the movie) or to the background.

To avoid user bias, the threshold is selected via an automated procedure. It initially analyses a small ROI of the movie with different thresholds and identifies the one that produces the best clustering. The silhouette score – a commonly used metric in clustering algorithms‐ evaluates the clustering quality.^[^ ^50^ ^]^ The silhouette score is calculated for each pixel by comparing two values, the internal correlation and the external correlation. The internal correlation is the average correlation of the pixel under evaluation with other pixels belonging to the same cluster. The external correlation is the highest correlation the pixel under evaluation has with pixels belonging to the nearby clusters. The silhouette score is the difference between the internal and external correlations, divided by the highest among them. It tells how well the pixel fits into its assigned cluster. The mathematical definition and more detailed explanation of the silhouette score are outlined in Note SIII (Supporting Information).

CLIM gives the following imaging contrasts:

1. The correlation map (Figure 1f) presents the average correlation of each pixel with its direct neighbors. The correlation map reveals the high and low correlation areas of the sample unrecognizable in the time‐averaged PL image (compare Figure 1a and Figure 1f).
2. The cluster map (Figure 1g) illustrates how the algorithm divides the image into clusters of highly correlated pixels. This allows us to see the shape and size of the clusters (or functional domains) and they can be used as ROI to calculate the time‐dependent integrated PL intensity of each cluster. The latter can be further analyzed to reveal time‐dependent PL fluctuation dynamics.
3. The internal correlation (Figure 1h) displays the average correlation each pixel has with other pixels of the same cluster. This aids in assessing the quality of the clustering and is necessary to calculate the silhouette score (see below).
4. The silhouette map (Figure 1i) shows how significantly more correlated a pixel is to a pixel within the same cluster compared to pixels in the nearby clusters. A score close to zero indicates that the pixel is not significantly more correlated with its assigned cluster when compared to its correlation with pixels from the nearby clusters. A score close to unity indicates a highly independent behavior meaning that the pixel is only highly correlated to its assigned cluster and not with the other nearby clusters. In addition to providing the assessment criterion for clusters’ quality, the silhouette map reveals information on inter‐cluster correlations, which can be linked to communication between clusters.

Because these contrasts are generated from intensity fluctuations inherent to the material, they are directly related to the photo‐induced process in the material, providing a functional mapping of these processes at the microscale.

Since CLIM is a novel imaging technique, it is essential to characterize its resolution and noise sensitivity. These aspects are explored in Notes SIV and SV (Supporting Information). We treat the resolution of the correlation map and the cluster map separately— the correlation map is a continuous signal, while the cluster map is binary. The cluster map's resolution was assessed through simulations, while the correlation map's resolution was evaluated using experimental data, discussed in the results section.

For the cluster map, we estimated the resolution by simulating two independent emitters (blinkers) moving closer together. Using the Houston resolution criterion,^[^ ^51^ ^]^ which defines the diffraction limit as the full width at half maximum (FWHM) of the point spread function (PSF), we examined distances ranging from 0.1 to 2 times the diffraction limit. We observed the ability of CLIM to separate the emitters. We also varied the PSF sampling rate by using different pixel widths (3 to 20 pixels). The results, detailed in Note SIV and Figure S4.1 (Supporting Information), indicate that the cluster map achieves a resolution of about half the diffraction limit, which is unaffected by the sampling rate.

Finally, we note here that CLIM is effective even for weakly fluctuating signals, requiring luminescence fluctuations just twice the standard deviation of the noise (Note SV, Supporting Information).

### 2.2 CLIM of MAPI Films

To showcase the capabilities of CLIM, we recorded PL movies of three methylammonium lead iodide (MAPbI_3_ or MAPI) thin films employing a standard widefield PL microscope (see Experimental Section for details). The MAPI films with different grain sizes ranging from 0.5 to 3 µm were fabricated following the previously developed recipes.^[^ ^47^, ^52^ ^]^ These films are labeled as follows: the porous film with very large grains (≈3 µm, MAPI‐POR), the film with large grains (≈1 µm, MAPI‐LG), and the films with small grains (≈0.5 µm, MAPI‐SG). The two latter films are specifically designed for efficient solar cells.^[^ ^52^ ^]^

**Figure 2:**
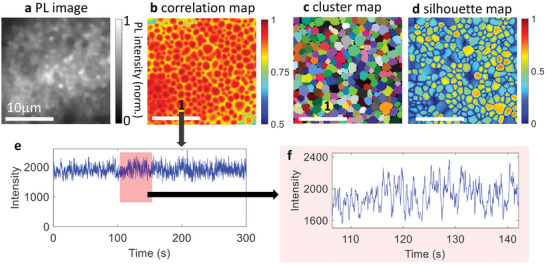
CLIM of MAPI‐LG film. a) PL image time‐averaged over the whole movie (Movie S3, Supporting Information), b) correlation map showing microscale domains of highly correlated pixels separated by boundaries comprised by pixels with lower correlations. c) cluster map obtained at 0.7 optimum threshold, where each color designates an individual cluster. The repeated colors visible in the image are due to the large number of clusters to be depicted in comparison with the available colors and thus do not indicate similarity. d) Silhouette map. e) Exemplary PL trace showing time‐dependent emission behavior of a cluster (marked as 1), and f) magnified view of the same trace. Scale bar = 10 µm. Samples were excited with a 488 nm diode laser in continuous wave mode and emission was collected from the entire PL band of the MAPI‐LG film.

**Figure** **2**a shows typical spatial heterogeneity in PL images of the MAPI‐LG film; for the other films, see Note SVI (Supporting Information).^[^ ^19^, ^20^, ^21^, ^23^ ^]^ We found that local PL intensity substantially fluctuates in all studied films when measured at ambient conditions, see Movies S2–S4 (Supporting Information).^[^ ^41^ ^]^ Although substantial PL fluctuations or blinking are mostly reported for isolated nanocrystals and microcrystals,^[^ ^33^, ^34^, ^35^, ^53^ ^]^ here we show that this can be observed even in thin films with grains as large as 3 µm, indicating the universality of the PL fluctuation phenomenon for MHPs. The presence of local PL fluctuations allowed us to use CLIM for these samples.

Figure 2 shows CLIM images of the MAPI‐LG sample; see also Note SVI (Supporting Information). First of all, significant differences between the PL image (time‐averaged) and the CLIM images (correlation, cluster, and silhouette maps) are observed, as also shown by the overlay of PL and all CLIM images in Note SVII (Supporting Information). The correlation map (Figure 2b) displays microscale regions of various sizes and shapes with high correlation at their centers, separated by low correlation boundaries. The cluster image reveals the same film as an array of microscale clusters or functional domains (Figure 2c). All pixels in each cluster show a correlation of at least 0.7 (the threshold used) with one another, and the average inter‐cluster correlation is <0.3. Although the correlation map does not show significant differences between clusters, the silhouette map across the same area displays significant heterogeneity at the scale above the cluster sizes. The silhouette metric typically ranges from 0.1 to 0.8 among clusters, and it is also low at the cluster boundaries. A low silhouette metric in a cluster means that there is a certain correlation (below the threshold, 0.7) between PL fluctuations in this cluster and PL fluctuations in the other clusters. Importantly, the spatial PL heterogeneity observed in the PL image (Figure 2a) does not always align with the clusters. Overlaying of the cluster contours and the PL image shows that a bright PL spot can be shared among several clusters (Note SVII and Figure S7.1c, Supporting Information), and that some of the large clusters possess inhomogeneous PL. Therefore, apparent domains in the heterogeneous PL image do not necessarily represent functional domains in perovskite films, highlighting CLIM's importance in understanding these systems' local photophysics.

Since the contrasts provided by CLIM are derived from the spatiotemporal PL heterogeneity, the CLIM maps visualize the sample's microscale heterogeneity of the optoelectronic properties. To obtain a more detailed picture, it is crucial to understand the relationship of the obtained microscale functional domains with the grain structure revealed in SEM images.

### 2.3 Comparison of SEM Image and CLIM Maps

**Figure 3:**
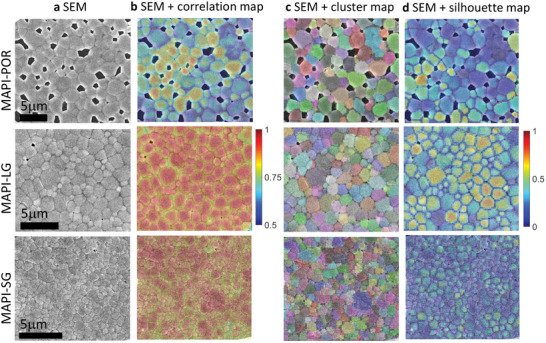
Comparison of CLIM maps and SEM for MAPI films of different grain sizes. a) SEM for MAPI‐POR, MAPI‐LG, and MAPI‐SG, b) SEM images of the corresponding samples overlayed with the correlation maps, higher correlations at the center of the grains are observed. c) SEM images overlayed with the cluster maps showing excellent matching of each grain with the corresponding individual cluster, and d) SEM images overlayed with silhouette maps. It shows that some grains present more independent fluctuations (high silhouette scores) while others have some degree of correlation leading to a lower silhouette score.

We imaged the same areas using scanning electron microscopy (SEM) to compare the clusters revealed by CLIM with the film microstructure. The overlays of the SEM images and the CLIM maps demonstrate striking similarities in the shape, size, and spatial arrangement of the clusters with the grains in the MAPI‐POR and MAPI‐LG samples (**Figure** **3**), with the cluster boundaries generally coinciding with the grain boundaries. To the best of our knowledge, this is the first time that such a clear morphological grain distribution commonly observed in SEM has been obtained through fluorescence microscopy for semiconductor films, establishing CLIM a highly valuable noninvasive tool for imaging semiconductor systems.

For the MAPI‐SG sample, matching between the SEM and CLIM imaging is less precise. This is due to the grain sizes approaching the resolution limit of our technique. Consequently, grains that are larger than the resolution limit are individually resolved, while smaller grains are not, leading to multiple grains being grouped into a single cluster. We used the sharpness of the boundary to estimate the resolution. If two grain boundaries are closer than this limit, they become indistinguishable. To estimate this, we analyzed the image complement of the correlation map (defined as one minus the correlation map), where the boundaries appear bright (Figure S4.2a–c and Note SIV, Supporting Information). A line profile across the boundary yielded a resolution of ≈600 nm, aligning with the theoretical diffraction limit of the system. Additionally, a decorrelation analysis for evaluating resolution based on Descloux et al.^[^ ^54^ ^]^ provided a similar resolution of 570 nm. While the resolution of the correlation map is not significantly higher than the diffraction limit, it resolved the boundary where the standard wide‐field microscopy PL imaging failed, and the cluster map still yielded a resolution improvement of a factor two (Note SIV, Supporting Information).

A comparison of the SEM map with the correlation map and silhouette map shows that lower correlation and silhouette coefficient values are observed at the grain boundaries, with a gradual increase toward the center of the grains. The correspondence between the grains and clusters, along with the high correlation between pixels within clusters (>0.7), suggests that the PL of most grains fluctuates independently.

However, the presence of grains with low silhouette scores (0 to 0.3) suggests that not all grains are entirely independent, indicating some degree of similarity between the signals of neighboring grains. This similarity, which can be quantified from the silhouette scores, likely stems from a certain degree of communication, resulting in partially similar PL fluctuations.

### 2.4 Extracting and Understanding PL Fluctuation Kinetics with CLIM

Insight into the nature of the nonradiative recombination (NR) centers controlling photophysical properties can be obtained from the analysis of PL intensity fluctuation kinetics.^[^ ^36^, ^53^, ^55^, ^56^, ^57^, ^58^ ^]^ However, in a polycrystalline film, it is difficult to predict the correct size, shape, and location of the areas (or a ROI) to extract the intensity traces. Clusters or functional domains obtained from CLIM are the natural ROI for extracting PL intensity traces and subsequent analysis of the PL fluctuation kinetics.

Since the CLIM clusters correspond to individual grains in the MAPI films studied here, analysis of PL intensity kinetics can give further insight into the nature of the fluctuating NR centers controlling photophysical properties in each grain.

**Figure 4:**
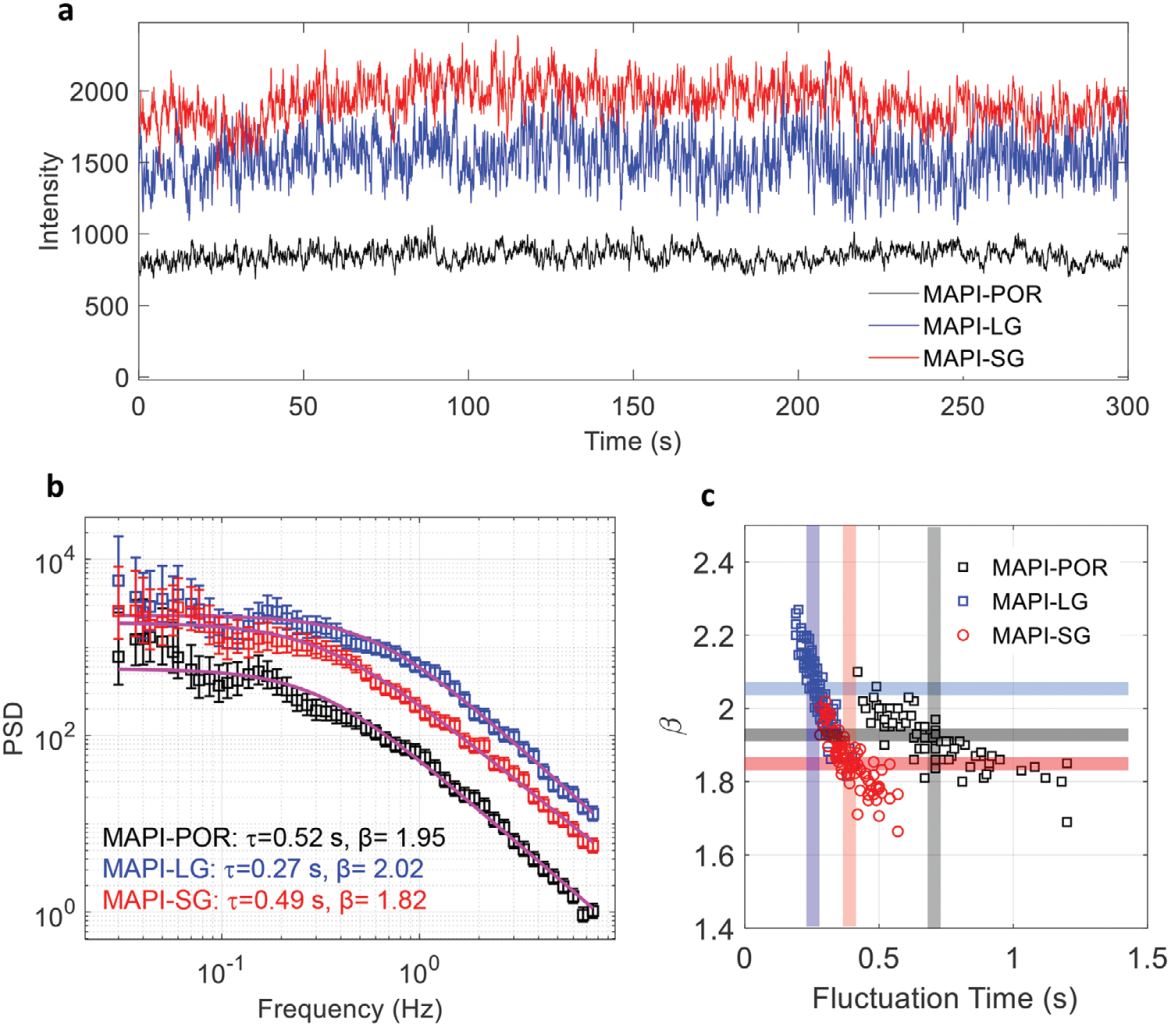
a) Representative PL traces and b) the power spectral density (PSD) curves of individual clusters obtained from MAPI‐POR, MAPI‐LG, and MAPI‐SG films. PSDs are fitted with stretched Lorentzian functions (Equation (1)). c) Statistics of the fitting parameters for all three samples. Average values of the stretching exponent (β) and the characteristic fluctuation time (τ) are shown by the faded lines of the same colors as the data points.

For instance, the presence per grain of only one fluctuating NR center which switches between an active and an inactive state makes the PL trace appear as a “telegraph signal” with distinct on (high PL intensity) and off (low PL intensity) levels.^[^ ^36^, ^55^, ^56^ ^]^ However, as the number of NR centers increases and their properties vary, it becomes difficult to identify clear intensity levels. This is the case for all samples studied here (**Figure** **4**a).^[^ ^34^, ^57^ ^]^ Therefore, the conventional analysis of PL fluctuation kinetics employing probability distribution of high and low‐intensity levels is not applicable here.

Alternatively, one can calculate the power spectral density (PSD) of the fluctuating PL signal, enabling unbiased analysis of the PL blinking data (see Note SVIII, Supporting Information).^[^ ^53^, ^59^ ^]^ Depending on the fluctuation behavior, the shape of the PSDs can vary from a Lorentzian function (indicative of a single type of switching center with a characteristic switching rate) to a power law. The origin of the latter is still debated,^[^ ^58^ ^]^ but it can be explained by the presence of multiple NR centers with switching rates spanning several orders of magnitude.

Figure 4a shows exemplary PL traces of individual clusters from three different MAPI films. Similar to the observations in individual sub‐micrometer MAPI crystals,^[^ ^53^ ^]^ PSDs of all these PL traces tend to saturate at low frequencies (<1 Hz), indicating the presence of a maximum characteristic timescale for these fluctuations (Figure 4b). The high‐frequency range resembles a power law with the slope (β) close to 2 indicating a Lorentzian PSD. This generic behavior known from individual crystals^[^ ^53^ ^]^ holds for most of the analyzed traces across all three samples: MAPI‐LG, MAPI‐SG, and MAPI‐POR, and can be described by a stretched Lorentzian function, see Equation (1) in the Experimental Section.

Statistical analysis of PSDs of more than 70 PL traces is shown in Figure 4c. The average characteristic time τ for MAPI‐POR is approximately twice as large as for MAPI‐LG and MAPI‐SG. The average β slightly decreases from MAPI‐LG to MAPI‐POR and MAPI‐SG. However, for all samples, the average β remains close to 2, suggesting that PL fluctuations are primarily controlled by one specific type of metastable NR centers with a characteristic switching time in the range of 0.2‐1.5 s. The β and τ values obtained from the fitting can be used as imaging contrasts. This way, we generate maps of the MAPI‐LG sample demonstrating the spatial distribution of the parameters of PL fluctuation kinetics (Note SVIII and Figure S8.2, Supporting Information).

To rationalize the parameters of the PL traces and PSD, we simulated the traces considering multiple (30‐50) two‐level systems (NR centers) per cluster with specific switching rates as detailed in Note SIX (Supporting Information). The simulated traces can reproduce the experimental ones rather well. Therefore, we conclude that several fluctuating NR centers with similar switching timescales control the photophysical properties of the functional structures or grains in MAPI films.

### 2.5 Imaging of the MAPI Layer in a Complete Solar Cell Under Operation

**Figure 5:**
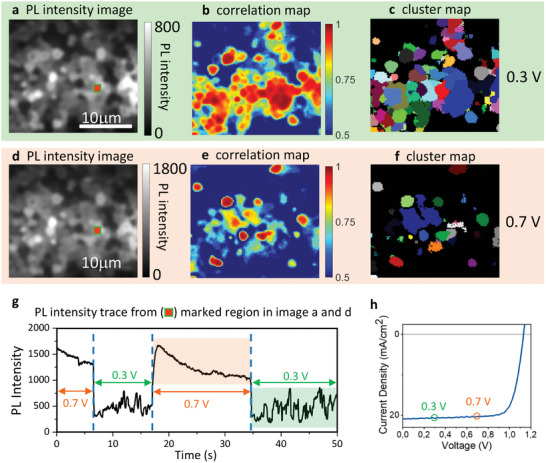
CLIM of a high‐efficient MAPI solar cell: PL images (movies) of a local area of the cell were recorded during sequential switching between two experimental conditions (0.3 and 0.7 V), with each condition maintained for 5 to 20 s. These movies showing local PL fluctuations were utilized to create CLIM images of the solar cell. a) average PL images, b) CLIM correlation map and c) CLIM cluster map obtained from a specific place of the solar cell under 0.3 V; d) average PL images, e) CLIM correlation map, and f) CLIM cluster map obtained from the same place under 0.7 V. g) PL intensity trace of the region marked by the red square in the PL images. PL intensity behavior changes in correlation with applied voltage (shown by labels in the figure). The CLIM images were generated using the PL movies recorded during the time intervals highlighted in g) by light green and light orange. h) Current density–voltage (J‐V) curve of the solar cell (power conversion efficiency −18.5%). The green and orange circles mark two experimental conditions used for the CLIM analysis. The device MAPI layer was excited at 480 nm with a power density of 0.15 W cm^−2^.

We applied PL microscopy to inspect a solar cell under operation with the MAPI‐LG film as an active layer (**Figure** **5**). We consecutively switched between 0.3 and 0.7 V after allowing tens of seconds in each condition. As expected, the PL intensity was ≈50 times lower than for the corresponding MAPI film due to efficient charge extraction by the electrodes. PL of the cell appeared much more spatially inhomogeneous than that of the film. Surprisingly, very large fluctuations in the local PL intensity were observed. These fluctuations had much larger relative amplitudes (compare Figures 4a and 5b for 0.3 V) than the PL fluctuations in the MAPI‐LG film alone (discussed before).

The dependence of the local PL fluctuations on the voltage is illustrated in Figure 5 where PL from a local region of the device is monitored. The largest fluctuations are observed for V < 0.4V. Upon increasing the voltage above 0.7 V, PL becomes stronger, more homogeneous, and less fluctuating but still shows some dynamics. At V > 1.2 V, the total emission of the device consists of both PL and electroluminescence (Note SXI and Figure S11.3, Supporting Information). These observations were repeatable when the voltage was scanned up and down (Figure 5g).

The presence of fluctuations allowed us to obtain clear CLIM images of the solar cell, as shown in Figure 5a–f). The appearance of the images is different for low (0.3 V) and high (0.7 V) voltages because the voltage influences not only the amplitude but also the location of the fluctuating regions. The clusters revealed by CLIM (regions with highly correlated PL intensity dynamics) in most cases are much larger (several µm) than the film grains (≈1 µm), which makes these images quite different from those of the bare MAPI‐LG films (compare Figure 2 with Figure 5). It is to be noted that direct comparison of CLIM and SEM images is possible only in bare MAPI films and not in multilayer devices, where CLIM is the only means to resolve microscale clusters. However, comparing CLIM images of similar MAPI films on a glass substrate and inside a device is an indirect method of identifying the relationship between CLIM clusters in a solar cell and expected morphological grain distribution.

## 3 Discussion

The results presented above demonstrate that CLIM can be applied to both thin films of MHPs and functioning devices – solar cells, in our case. This capability arises from the dynamic nature of MHPs, evidenced by their PL fluctuation/blinking in individual nano and microcrystals and within local regions of thin films and perovskite layers in functioning devices. These observations suggest that CLIM visualizes the intrinsic dynamics of MHPs, including ion migration, defect creation and annihilation, and charge generation and extraction processes in solar cells at the microscale.

In MAPI films, the structures revealed by CLIM are almost identical to the grain morphology observed in the SEM images of the same areas of the film. This means that the material dynamics in the polycrystalline film differ from grain to grain and that the neighboring grains, at first approximation, do not significantly influence each other. In essence, if one grain experiences an efficient quenching leading to a decrease in its PL intensity, this has little to no impact on the PL of the adjacent grains.

However, a more detailed analysis reveals some extent of “communication” between the grains, evidenced by correlation in fluctuations observed through the silhouette contrast (Figure 3d) where some grains show high silhouette (fluctuations of their PL are highly individual) while the others – low silhouette (presence of some similarities in their PL fluctuations). This communication likely indicates inter‐grain lateral charge carrier and/or ion diffusion. When the communication between several grains is strong, these grains belong to the same cluster in CLIM. Individual clusters do not necessarily align with the apparent structural boundaries observed in SEM images. While occasionally observed in films, this phenomenon was most strikingly revealed in functioning solar cells, even though the studied solar cell contains the polycrystalline MAPI film prepared using the same procedure as the MAPI‐LG film on the glass substrate. The difference in the microscale behavior of the PL fluctuations evidenced by CLIM must stem from the presence of the transport layers, electrodes, bias voltage, or a combination of these factors within the solar cell.

PL fluctuations in large nanocrystals and microcrystals can be well explained by the supertrap model,^[^ ^27^, ^37^ ^]^ where the activation and de‐activation of just one (or very few) efficient NR recombination centers (supertraps) cause fluctuation of the PL of the entire crystal.^[^ ^27^ ^]^ Our computer simulations (Note SIX, Supporting Information) show that the PL fluctuation traces and PSDs of individual grains in MAPI films can be well mimicked in the framework of this model. To do so, we needed to make a rather reasonable assumption of the number of fluctuating NR centers per grain, which linearly scales with the grain volume (for a given concentration of NR centers). The presence of numerous NR centers per a considerably large grain makes the relative amplitude of the PL fluctuations rather small (15–30% relative fluctuation amplitude, consistent with our experiments) due to the ensemble averaging effect.

Furthermore, if we consider that the solar cell's fluctuating regions (clusters) are even larger than those in the film, we expect PL fluctuations amplitude to be even smaller. Contrary to this expectation, very large clusters in the solar cell exhibited very large fluctuation amplitudes. Moreover, these fluctuations usually did not appear abruptly, as semi‐continuous increases and decreases in intensity were observed over time (see Note SXI, Supporting Information). Considering all these, we conclude that PL fluctuations in the solar cell cannot be explained by the presence of metastable NR centers (the super‐trap model),^[^ ^27^ ^]^ and other mechanisms of PL fluctuations must be considered.

The main PL quenching mechanism in solar cells is the extraction of photogenerated charge carriers by the contacts. It is well‐known that electron and hole transport from the light absorbing layer (MAPI in our case) are influenced by various chemical and physical processes at the interfaces.^[^ ^60^, ^61^, ^62^ ^]^ Therefore, fluctuations of charge extraction efficiency (or the contact “quality”) are likely to be the reason behind the observed local PL fluctuations.

Because the CLIM clusters in the solar cell usually contain several grains, the contact quality for all these grains should change synchronously. This implies the existence of strong “communication” between the grains comprising the cluster. For example, this can occur via a lateral charge carrier transport between the grains (inter‐grain communication).^[^ ^34^, ^63^, ^64^ ^]^ We hypothesize that a local random opening of a very efficient charge extraction channel in one of the grains causes variation of the charge extraction in all cluster grains, making them fluctuate synchronously. Alternatively, changes in the properties of the interface between the cluster and the electrode, such as local charging/discharging, may also be responsible for synchronous fluctuations.

The dependence of the PL fluctuations on the applied bias voltage is the key observation to consider to reveal the nature of the PL quenching instability. Note that it is not the current density but the bias voltage that is important because at both 0.3 and 0.7 V, the current remains similar (refer to the *J–V* curve in Figure 5h), yet the temporal PL behavior differs significantly. From this, we deduce that additional metastable PL quenching channels (likely charge extraction channels) become obstructed at high bias. Consequently, we observe stabilization (from fluctuations) of the PL intensity and its increase. Higher bias voltage may lead to a higher interfacial charge density, suggesting that these metastable charge extraction channels deactivate due to the increased charge density between the perovskite layer and the contacts. Further experiments are needed to rationalize the nature of the metastable PL quenching behavior in the MAPI solar cells.

Although fluctuations at bias voltages above 0.7 V PL were infrequent, PL was still not constant. It exhibited rapid initial enhancement followed by gradual bleaching dynamics (Figure 5g). These gradual processes are most likely associated with ion migration that changes the device's energetic and electric field profiles as the ions redistribute, ultimately reaching a steady state after several seconds. Note that if these dynamics are similar across several image pixels, CLIM assigns these pixels to one cluster. That is why even at 0.7 V, when the fluctuations are minimal, some clusters are still observed. Therefore, clusters always reveal regions with correlated dynamics, whether it is blinking‐like behavior or gradual changes.

As a fully optical, non‐invasive method, CLIM can image luminescent materials even when covered by other layers, including electrical contacts, as long as these layers are transparent to light. The closest method to CLIM is PL microscopy imaging and spectroscopy because both techniques look at the same signal.

Despite this, CLIM employs a completely different approach to obtaining imaging contrasts. CLIM analyzes the statistics of spatially and temporally resolved (resolution of wide‐field imaging is in the ms range) PL intensity dynamics over a defined time interval (e.g., seconds) to extract imaging contrasts. Traditional PL microscopy generates image contrasts directly from PL properties (intensity, spectrum, and lifetime) averaged over specific acquisition times. Therefore, CLIM utilizing spatiotemporal correlations is complementary to the traditional PL microscopy and spectroscopy. For example, local material restructuring,^[^ ^65^ ^]^ aging/degradation,^[^ ^66^ ^]^ chemical reactions, ion migration,^[^ ^67^ ^]^ and other phenomena common for MHPs^[^ ^68^ ^]^ create heterogeneities and spatiotemporal correlations mostly hidden for traditional PL microscopy but readily revealed by CLIM. Understanding these processes is crucial for material quality control, optimizing device performance, and ensuring stability.

The reader may find conceptual parallels between CLIM and Super‐resolution Optical Fluctuation Imaging (SOFI)^[^ ^17^ ^]^ that is also based on the correlation of intensity fluctuations. The principal difference between CLIM and SOFI is in the purposes for which these methods were developed. SOFI was developed to increase spatial resolution, while CLIM is designed to extract local photophysical information where resolution improvement comes as a bonus. In this context, the CLIM cluster map (just one of many CLIM contrasts) with a 2 times resolution enhancement yields a similar resolution improvement as the second‐order SOFI. The standard SOFI algorithm is designed to apply to structures labeled with dye molecules possessing a similar brightness and blinking dynamics – the condition that cannot be realized in luminescence materials. Even if balanced SOFI can potentially solve that problem,^[^ ^69^ ^]^ SOFI can only extract blinking dynamics by using assumptions and/or specific models for the underlying dynamics^[^ ^70^ ^]^ and, therefore, it has clear limitations for this type of application. The advantage of CLIM is that it enables the clustering of pixels and subsequent extraction of PL dynamic response without any pre‐assumptions or models. Additionally, the CLIM clustering offers comprehensive analyses, such as inter‐cluster correlations and cluster sizes and shapes, which are not easily achieved with the SOFI images. Therefore, CLIM appears more suitable for samples presenting inhomogeneous behavior, which may change over time and where functional information is crucial, such as semiconductors and solar cells.

The main advantage of CLIM lies in revealing contrasts originating from properties previously unused for imaging purposes. Although the clusters in the studied MAPI films correspond to grains, they might not follow the same trend for other material systems. Nevertheless, CLIM will identify functional domains if the material possesses nano/microscale changes in PL (fluctuation, enhancement, bleaching). By combining this with SEM, CLIM reveals structure‐function relations, offering unique opportunities to understand materials and devices. Today, fluctuations of PL are commonly observed in many contemporary material systems (including 2D materials),^[^ ^38^, ^39^, ^40^ ^]^ so we anticipate that CLIM will have broad applications in both fundamental and applied sciences.

## 4 Conclusion

We developed a noninvasive functional imaging method—Correlation Clustering Imaging (CLIM)—that exploits the correlation of PL fluctuations in space and time. CLIM provides a spatially resolved view of photophysical processes in dynamic luminescent materials, such as the formation and annihilation of PL quenching channels and lateral communication inside and between structural nano‐ to microscale regions. The method can be applied to any luminescent sample, including in operando devices (solar cells, light emitting diods, field effect transistors, etc.), as long as the PL shows local dynamics over time.

Metal halide perovskites are particularly suitable for the application of CLIM. We demonstrate that CLIM often produces images similar to those from SEM, suggesting that noninvasive CLIM can replace the more invasive SEM for resolving the microstructure of polycrystalline films. While PL fluctuations in perovskite films have a similar photophysical nature to those in individual crystals, the discovered local PL dynamics in perovskite solar cells is a novel phenomenon with a completely different nature. We hypothesize that these dynamics are due to processes at the interfaces with transport layers, creating microscale transient PL quenching channels sensitive to applied voltage.

Due to the ease of the method, we anticipate applying CLIM at various fabrication stages of perovskite devices to monitor the evolution of the materials and the impact of individual layers on the microstructure and function. Based on the facts, we foresee CLIM, with its novel imaging contrasts, becoming an important technique enabling unique insights into the microstructure‐function relationship in dynamic materials and devices.

## 5 Experimental Section

### Sample Preparation—Thin Film Fabrication

Methylammonium iodine (MAI) and lead acetate trihydrate Pb(Ac)2·3(H2O) (at 3:1 molar ratio) were dissolved in anhydrous N,N‐dimethylformamide (DMF) with a concentration of 40 wt.% with the addition of hypophosphorous acid (HPA) solution (1.5, 8 or 14 µL/1 mL DMF). The different amounts of HPA made it possible to control the grain size and porosity of the layers based on previously developed methods.^[^ ^52^ ^]^ The perovskite solution was spin coated at 2000 rpm for 60 s in a drybox (RH < 0.5%). After spin coating, the samples were dried 20 s by a stream of dry air. Afterward, the samples were kept at room temperature for 5 min and subsequently annealed at 100 °C for 5 min.

### Sample Preparation—Photovoltaic Device Fabrication and Characterization

Prepatterned indium tin oxide (ITO) coated glass substrates (PsiOTech Ltd., 15 Ohm sq^−1^) were ultrasonically cleaned with 2% hellmanex detergent, deionized water, acetone, and isopropanol, followed by 8 min oxygen plasma treatment. Poly(triarylamine) PTAA prepared based on the previous report^[^ ^52^ ^]^ was spin‐coated on the clean substrates with 4000 rpm for 30 s and annealed at 100 °C for 10 min. After the deposition of the active layer (see above) the samples were transferred to a nitrogen‐filled glove box, where phenyl‐C61‐butyric acid methyl ester (PCBM) (20 mg mL^−1^ dissolved in chlorobenzene) was dynamically spin coated at 2000 rpm for 30 s on the perovskite layer followed by a 10 min at 100 °C annealing. Sequentially, bathocuproine (BCP) (0.5 mg mL^−1^ dissolved in isopropanol) or the π‐extended phosphoniumfluorenes (0.5 mg mL^−1^ dissolved in methanol) was spin‐coated on the PCBM, forming a thin layer ≈5 nm and 10 nm, respectively. To complete the device, 80 nm silver was deposited via thermal evaporation under high vacuum. The *J–V* curves were measured under an illumination of AM 1.5 sunlight with 100 mW cm^−2^ irradiation from an Abet Sun 3000 Class AAA solar simulator. The spectral mismatch factor corrected the light intensity before testing. The measurements were conducted in air without encapsulation.

### Photoluminescence Measurements

A home‐built wide‐field inverted fluorescence microscope was employed to measure perovskite film PL. The samples were excited using a 485 nm diode laser (PicoQuant, continuous wave mode), guided through the IX‐71 (Olympus) microscope body and a 40× dry objective with NA = 0.6. A set of neutral density filters was used to achieve an excitation power density of 0.15 Wcm⁻^2^. The PL signals were collected through the same objective, filtered through two long‐pass filters (500 and 680 nm), and recorded using an EMCCD camera. The excitation spot size at the sample plane measured 30 µm in diameter, and each pixel corresponded to 200 nm × 200 nm area on the sample. Each PL fluctuation movie, with a duration of 5 minutes, consisted of 6000 frames recorded with a 50 ms exposure time.

### Scanning Electron Microscopy Measurements

SEM imaging used a Hitachi SU8010 cold field emission scanning electron microscope. The samples were mounted on a standard SEM holder using a copper tape. To facilitate high‐resolution imaging and prevent sample charging, a thin layer of conductive metal alloy (Pt:Pd = 80:20) was coated on the sample. Images were captured using an In‐lens secondary electron detector at accelerating voltages ranging from 5 to 15 kV.

### Precise Aligning Optical and SEM Images

For a precise comparison of the structure and optoelectronic response of the films, it was crucial to achieve a sharp alignment of the SEM images, PL images, and CLIM maps. Thin films were marked with careful scratching and by local burning the sample with a high‐intensity laser. These marks were correlated with the regions of interest studied via PL microscopy by concurrently recording light transmission images. The PL measurements were conducted away from the scratches and any debris resulting from scratching. Optical images were corrected for drift, and the overlay of optical and SEM images was accomplished by scaling the images, followed by thier translation and rotation.

### CLIM Algorithm for Functional Imaging

The algorithm is explained in detail in Note SIII (Supporting Information) and the code is available here: https://github.com/BorisLouis/CLIM/releases/tag/CLIMv1.0.

### Power Spectral Density Estimation of Photoluminescence Fluctuations and Fitting

Details of the theory and algorithm of the power spectral density estimation are provided in Note SVIII (Supporting Information) and previously published work^[^ ^53^, ^58^ ^]^ and the code is available,^[^ ^71^ ^]^ (https://doi.org/10.5281/zenodo.8027381). In emitting semiconductor systems, PL fluctuation results from the combined response of several types of NR recombination processes, which can be modelled as a combination of multiple two‐level systems (TLSs). Depending on whether the contribution arises from one or several types of TLSs with significantly different switching rates, the PSD can exhibit a range of signatures, spanning from pure Lorentzian to stretched Lorentzian (as described by Equation (1)) to pure power‐law behavior (as described by Equation (2)), each with variable slopes.

(1) $$PSD\;\left(f\right)=\frac{A}{\;1+{\left(f/f_{0}\right)}^{\beta }}$$

(2) $$PSD\;\left(f\right)=\frac{A^{\prime }}{\;f^{\beta }}\;$$

here ƒ_0_ = 1/(2πτ) and τ is the characteristic fluctuation time. β is the stretching exponent, equals 2 for fluctuations caused by one or several TLSs with the same characteristic frequency. In general the amplitude of PSD (the constants A and A’ in these specific cases) is proportional to the square of the mean signal and the square of the relative fluctuation amplitude Δ𝐼⁄𝐼𝑚𝑎𝑥, see ref.[53] for more details.

## Conflict of Interest

The authors declare no conflict of interest.

## Author Contributions

B.L. and S.S. contributed equally to this work. B.L., S.S., I.G.S., and J.H. conceived the project and planned the experiments with further input from Y.V., B.L. developed the CLIM algorithm with additional input from S.S., A.Q. fabricated the perovskite thin films. A.Q. and J.R. fabricated the devices and performed electrical characterization. B.L. and S.S. performed the PL microscopy measurements and analyzed the data. S.S. performed the SEM measurements and correlated them with CLIM output with additional help from B.L.Photovoltaic devices under operation were measured and power spectral densities analysed by S.S. with help of I.G.S. The manuscript was written by B.L., S.S., and I.G.S. with input from J.H. and Y.V.; All authors discussed the results and provided feedback on the manuscript.

## Supporting information



Supporting Information

Supplemental Movie 1

Supplemental Movie 2

Supplemental Movie 3

Supplemental Movie 4

Supplemental Movie 5

## Data Availability

The data that support the findings of this study are available from the corresponding authors upon reasonable request.
