# Supplementary material for: In Operando Locally‐Resolved Photophysics in Perovskite Solar Cells by Correlation Clustering Imaging
Source: Adv Mater. 2024 Dec 29;37(7):2413126. doi: 10.1002/adma.202413126 (PMC11837892; doi:10.1002/adma.202413126)
Supplement: Supplementary file 1 — Supporting Information [file ADMA-37-2413126-s002.pdf]

ADVANCED MATERIALS

Supporting Information

for *Adv. Mater.*, DOI 10.1002/adma.202413126

# In Operando Locally-Resolved Photophysics in Perovskite Solar Cells by Correlation Clustering Imaging

*Boris Louis, Sudipta Seth*, Qingzhi An, Ran Ji, Yana Vaynzof, Johan Hofkens* and Ivan G. Scheblykin**

**In Operando Locally-Resolved Photophysics in Perovskite Solar Cells by Correlation Clustering Imaging**

Boris Louis[1,2,+], Sudipta Seth[1,2,+,*], Qingzhi An[3], Ran Ji,[3,4] Yana Vaynzof [3,4], Johan Hofkens[2,5*], Ivan G. Scheblykin[1*]

[1] Division of Chemical Physics and NanoLund, Lund University, PO Box 124, Lund 22100, Sweden

[2] Laboratory for Photochemistry and Spectroscopy, Division for Molecular Imaging and Photonics, Department of Chemistry, Katholieke Universiteit Leuven, Leuven 3001, Belgium.

[3] Chair for Emerging Electronic Technologies, Technical University of Dresden, Nöthnitzer Str. 61, 01187 Dresden, Germany

[4] Leibniz-Institute for Solid State and Materials Research Dresden, Helmholtzstraße 20, 01069 Dresden, Germany

[5] Max Planck Institute for Polymer Research, Mainz 55128, Germany

[+] These authors contributed equally.

* Corresponding Authors

# Table of Contents

Supporting Movie .......... 3

Note SI – Microscopic in-operando solar cell measurement setup .......... 4

Note SII – Image simulations .......... 5

Note SIII – Correlation clustering imaging (CLIM) .......... 6

CLIM workflow description .......... 6

Pre-processing .......... 7

Step 1 – Correlation analysis .......... 7

Step 2 - Clustering .......... 7

Threshold Selection for Unbiased Clustering .......... 8

Note SIV – Resolution of the method .......... 10

Resolution of the cluster map .......... 10

Resolution of the correlation map .......... 11

Note SV – Influence of uncorrelated and correlated signals .......... 13

Uncorrelated signals .......... 13

Correlated signals .......... 14

Note SVI – CLIM on $MAPbI_3$ films with different grain sizes .......... 16

Note SVII – Overlay of CLIM outputs. .......... 17

Note SVIII – Analysis of PL fluctuation kinetics .......... 18

PSD mapping of the clusters .......... 20

Note SIX – Blinking simulations........................................................................................................21

Note SX – Size dependence of the β and τ values obtained from the stretched Lorentzian fitting of the PSDs of the traces from the same sample.........................................................................................23

Note SXI – Device structure, performances and CLIM imaging...........................................................24

Note SXII – Characteristics of PL Intensity jumps ..................................................................................26

References ...............................................................................................................................................27

## Supporting Movie

**Movie S1** – Simulated array of individually blinking emitter used for the development of the CLIM method.

**Movie S2-4** – PL microscopy movies of MAPI-POR, MAPI-LG and MAPI-SG films in ambient conditions

**Movie S5 –** PL microscopy movie of a solar cell device alternating between two operating conditions (0.3 V and 0.7 V).

## Note SI – Microscopic in-operando solar cell measurement setup

MAPI thin films were measured employing a home-built wide-field inverted fluorescence microscope as shown below. For the measurement of the solar cells, the same microscope setup is used. In-operando measurement conditions are created by connecting the solar cells with a source meter and exciting with one sun equivalent irradiation. Excitation was done with a 488 nm diode laser.

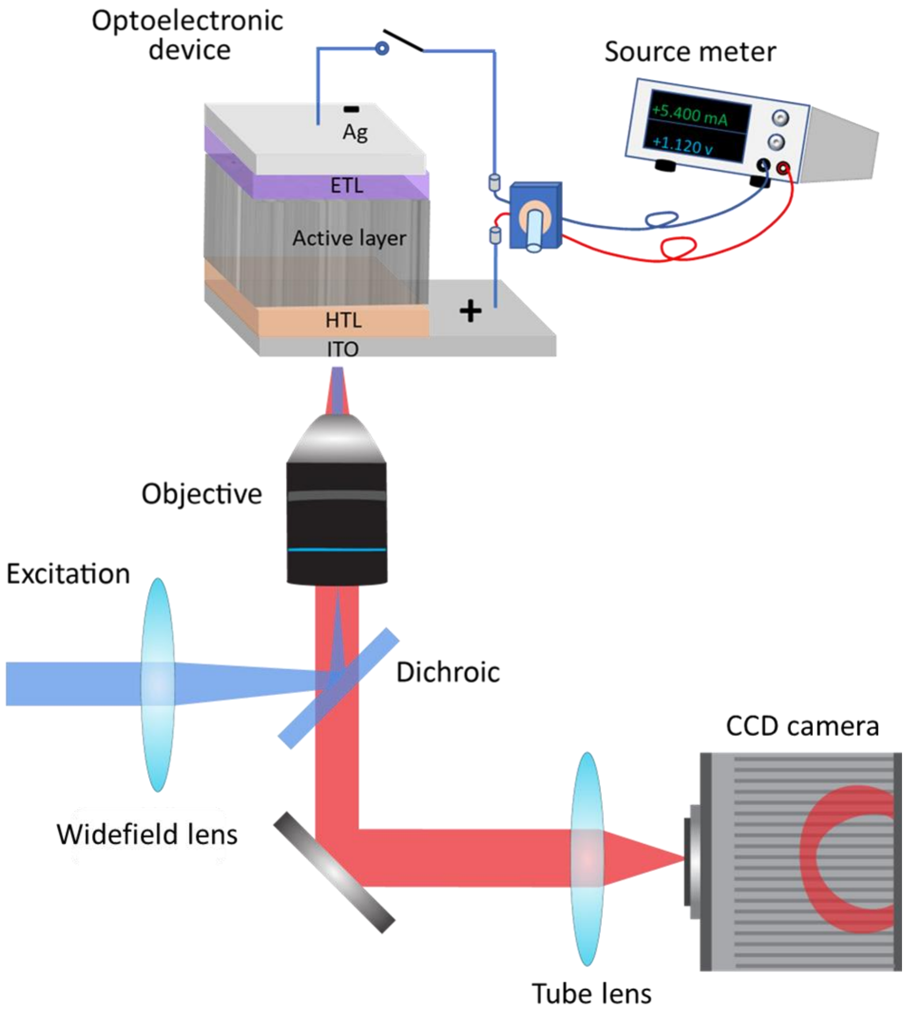


**Figure S1**. Schematic illustration of in-operando solar cell measurement setup based on a conventional widefield fluorescence microscope with CCD camera. Same microscope is used to measure MAPI films on the glass and MAPI solar cells under operation.

# Note SII – Image simulations

To evaluate and fine-tune the Correlation Clustering Imaging (CLIM) analysis, we employed simulated images to replicate the behavior of a blinking film. The simulation consists of three distinct components, as depicted in Fig. S1.

*1. Intensity Fluctuations*

We simulated intensity fluctuations as a two-level system with an equal probability of switching between the two levels (states). For the "ON" state (highest intensity), a base intensity was chosen (e.g. 1000 counts). The "OFF" state (lowest intensity) was calculated to be the base intensity multiplied by (1 – relative quenching efficiency). For this, in each time frame a random number between 0 and 1 was generated. If this number fell within the specified probability range (e.g. below 0.05 if a 5% switching probability was selected), a switch occurred from the current state to the other. To enhance the realism of the simulation, we generated the intensity traces at a higher time resolution and subsequently performed binning to account for the limited time resolution of the camera. Intensity traces were individually simulated for each of the 100 blinkers used to generate the image.

*2. Image Generation*

To generate realistic images, we had to consider the point spread function (PSF), for which we employed a 2D Gaussian distribution with a standard deviation of 3 pixels. The intensity of the Gaussian was normalized such that the maximum intensity equaled 1. For the simulation of a 10x10 array of blinkers, we placed a delta function at each desired blinker location. The intensity of the delta function corresponded to the simulated intensity of the blinker as determined in step 1, thus varying over time. Subsequently, the images of these time-dependent intensity delta functions were convolved frame by frame with the normalized PSF, resulting in the final movie. A representative image is presented in Fig. S2.1.

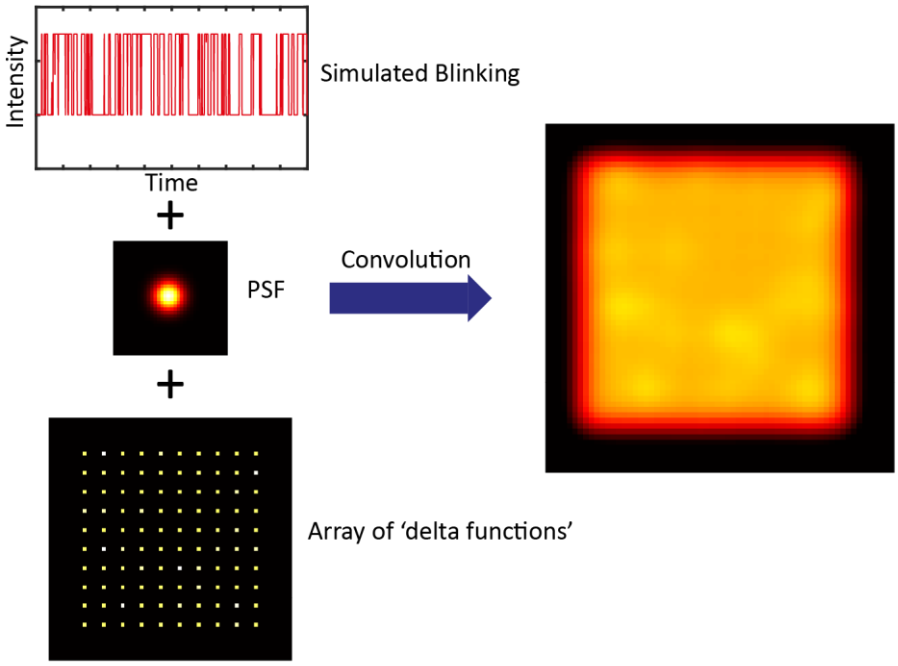


**Figure S2.1** Image simulations workflow. Images are simulated by combining simulated intensity traces with a 10x10 grid of delta functions whose intensity depend on the simulated traces. The 10x10 grid is then convoluted with the Gaussian PSF.

# Note SIII – Correlation clustering imaging (CLIM)

## CLIM workflow description

A schematic of the CLIM workflow is given in Scheme S3.1.

**Preprocessing**

1) Drift correction of the movie
2) Removal of background correlation using averaged time-dependent intensity over the whole image

**Step 1: Correlation analysis**

1) Scan movie pixel by pixel, and for each pixel:
   a) Compute Pearson correlation in time (intensity trace) with direct neighbors (lateral and diagonal yielding 8 values). See Figure 1b in the main text, for exemplary correlation ROI.
   b) Calculate average correlation to neighbors by averaging the correlation value obtained in 1a)
2) Make a list of all pixels in the image sorted by their average correlation to their neighbors (from highest to lowest) and keeping track of individual correlation values with their neighbors. This is the **list of potential seeding pixel**, for cluster growth
3) Cleanup the list, average correlation to neighbor below 0.2 is considered background and the corresponding pixels will not be further processed

**Step 2: Clustering**

**Note: Clustering is repeated on a small portion of the image (32x32 pixels) to find the optimal threshold, assessed using the higher average silhouette score over all the pixels. Then, the optimal threshold is applied to the entire image.**

Start from list of pixel generated in step 1:
1) If there is no pixels in the list, then analysis is done. Otherwise Select the pixel with highest average correlation to its neighbors to become the **seeding pixel**.
2) Add it to a new cluster
3) Look at the correlation of the seeding pixel to its neighbors;
   **if Correlation>threshold:**
   a) add these pixels to the cluster and remove them from the list of potential seeding pixels
   b) Look at the correlation of the freshly added pixels (to the cluster) with their neighbors
   c) If Condition 1* and Condition 2**
      are okay (→ back to 3a)
      **if no pixels fulfill the two conditions** (→ back to 1)

   *Condition 1: Correlation > threshold
   **Condition 2: Correlation with a random subset of pixels 10 pixels already in the cluster > threshold.

**Step 3: Output generation**

1) Intensity traces are calculated by integrating intensity over the pixels that belongs to the same cluster
2) Correlation map is generated from the average correlation to neighbors calculated in step 1, 1.b
3) Correlation to cluster is calculated by computing the average correlation of each pixels with all the pixels that belong to the same cluster
4) Silhouette is calculated for each pixels by:
   1) (b-a) / max(b,a)
   2) Where b is the correlation to cluster calculated in step 3.3
   3) Where a is calculated by computing the correlation between the intensity trace of the pixel treated and the average intensity trace from neighboring clusters

**Post processing**

1) Cluster size and shape can be extracted
2) Correlation between clusters can be computed
3) Kinetics of intensity fluctuations
4) Correlation between cluster, cluster size, shape, PL kinetics can be used as contrast to have a view on their location on the image and potentially correlate with structural feature

**Scheme S3.1**. Illustration of the workflow of the CLIM analysis developed in this work

## Pre-processing

Drift correction is achieved using image correlation through "normxcorr2" in MATLAB. Typically, very small drift occurs during measurements, around 1-2 pixels (200-400 nm) per 30 minutes.

To discard the background pixels from the analysis we compute the autocorrelation of each pixel. The background decays significantly faster than fluctuating signals allowing the background removal.

To eliminate the influence of signals that are correlated over the entire area (e.g. laser fluctuations), we calculate the spatially averaged intensity trace over all pixels in the image (excluding the background). Then, we deconvolve the time-evolution out of each pixel with the spatially averaged intensity trace to remove any correlation that affects all pixels uniformly.

## Step 1 – Correlation analysis

First, the movie is scanned in the xy-plane using a 3x3 correlation ROI. The correlation in time of each pixel in the ROI with the central pixel (cPx) is calculated (see Fig. 1a, main text). This means that we correlated the time-dependent intensity trace of the mentioned pixels. Note that when we will talk about the correlation between a pixel and another pixel or another quantity such as a cluster, we always correlate time-dependent intensity signals (from the pixel or the other quantity). Consequently, after this step, each pixel in the image (except those at the edges) has a set of 8 direct neighbors to which the correlation is known. Examples of such correlation ROI are given in Fig. 1b-d (main text) showing that some ROIs contain highly correlated pixels (green, Fig. 1b, main text), while others may contain a mix of high and low correlation values (Fig. 1d, main text) or just low correlation values (red, Fig. 1c, main text). Pixels with correlations to their neighboring pixels lower than 0.2 are removed from the analysis. The average correlation of each pixel to their neighbors (|NC|) is also computed and yields the correlation map shown in Fig. 1f in the main text.

At the end of this step, we have a list of all relevant pixels of the movie which we refer to as **the list of potential seeding pixels** (for cluster growth), **the correlation values to their 8 direct neighbors**, and the **correlation map** representing the average correlation with neighboring pixels (|NC| values) for each pixel of the image (Fig. 1f).

## Step 2 - Clustering

The clustering process is illustrated in Fig. 1e in the main text and briefly explained in Scheme S3.1. Essentially, it starts from the **list of potential seeding pixels**. The one with the highest average correlation to its neighbors (|NC| value) becomes a seeding pixel and therefore, is added to a new cluster. Then, the neighbors of the seeding pixel are inspected to see if their correlations to the seeding pixel are above a predefined threshold (see the dedicated section for explanation), if yes, they are also added to the cluster. The neighbors of the freshly added pixels are also inspected in the same way to grow the cluster. However, there are now two conditions to be fulfilled, this is to ensure cluster consistency: 1) The correlation of the neighbors of the freshly added pixels with the freshly added pixels needs to be above the threshold (the same as before), 2) the averaged correlation between the pixels that passed the first condition with a randomly selected subset of ten pixels that are already assigned to the cluster is calculated and needs to be above the threshold. We chose to compute on a subset of 10 pixels to have representative correlation values while limiting the computing time. At a certain point, the algorithm will not find any more pixels that fulfill the conditions. Then the cluster growth is ended, and the pixels assigned to it are removed from the list of potential seeding pixels. The algorithm restarts from the list of potential seeding pixels which has now less pixels than before and will repeat the procedure until all pixels are treated.

## Threshold Selection for Unbiased Clustering

To ensure the unbiased selection of the threshold, our algorithm autonomously determines the threshold. We execute the algorithm on a small portion of the image (32x32 pixels) while systematically varying the threshold from 0.4 to 0.9 in increments of 0.05. The optimal clustering result is chosen based on a specific metric to assess the quality of the clustering at each threshold. As metric, we used the "silhouette," a standard machine learning metric for evaluating the performance of clustering algorithms.[1] The equation for the silhouette is:

$$s(i) = \frac{b(i) - a(i)}{\max\{a(i), b(i)\}}, if\ |C_I| > 1 \qquad \text{(S3.1)}$$

$$s(i) = 0, if\ |C_I| = 1$$

Where i is the index of the pixel treated inside of cluster $C_I$. $|C_I|$ is the number of pixels belonging to cluster *I*.

$$a(i) = \frac{1}{|C_I| - 1} \sum_{j \in C_I, i \neq j} d_{int}(i,j) \qquad \text{(S3.2)}$$

a(*i*) describes the average correlation between pixels inside cluster *i*. $d_{int}(i,j)$ is equal to one minus the correlation between pixel i and j both belonging to cluster $C_I$.

$$b(i) = \max\ d_{ext}(i,K) \qquad \text{(S3.3)}$$

The term *b(i)* characterizes the largest average correlation that can be observed between pixels within cluster *I* and nearby clusters ($C_J$, $C_K$, $C_L$,…). To determine this value, we calculate the correlation between the pixels in cluster I (their time-dependent intensity trace) to all other clusters ($C_J$, $C_K$, $C_L$ and so on, taking the average time-dependent intensity trace of all pixels within the cluster) and retain the largest correlation value. In practice, we only calculate the correlation to the 10 closest clusters (in space) to reduce the computing time. $d_{ext}(i,K)$ is equal to one minus the correlation between pixel i from cluster I and the average time-dependent trace of all pixel in cluster K, for K = 1,2,…,10 indices of the 10 closest cluster to cluster I.

In simpler terms, the silhouette assesses how well an individual pixel fits within the cluster to which it has been assigned. It does so by comparing two quantities, denoted as **a** and **b** (Eq. S3.2 and Eq. S3.3 and Fig. S3.1 below). The parameter **a** represents the average correlation between the treated pixel and the pixels within the same cluster and is thus referred to as the internal correlation. Fig. S3.1a, shows two exemplary pixels (white square with black or blue border) within the yellow cluster, where the red disks indicate the pixel to which the correlation calculation is done. The red dots with a blue or black circle around it are only used in the calculation of parameter **a** for the pixel with blue or black border, respectively. In practice, the correlation with all pixels in the cluster is averaged to obtain the internal correlation. It is worth noting that due to crosstalk with the adjacent deep blue cluster (due to diffraction limits), the pixel with the black border (Fig. S3.1) will have a lower internal correlation compared to the pixel with the blue border which will likely have a purer signal from the underlying fluctuations.

The parameter **b** is computed by calculating the correlation between the treated pixel (its time-dependent intensity trace) and the averaged time-dependent fluctuations of the 10 closest clusters (calculated by averaging the time-dependent fluctuations of all pixels within the specified cluster). Out of the 10 correlation values obtained, we use the highest score as value for parameter b, which we refer to as external correlation. Fig. S3.1b illustrates the calculation of correlation to neighboring

clusters using red disks to show that here we are considering correlation to the entire cluster (using averaged time-dependent intensity over all pixels assigned to that cluster). By comparing the internal correlation, **a** and the external correlation **b**, we obtain the silhouette and gain insight into how well the pixel aligns with the cluster to which it has been assigned (see Equation S3.1). Note, we chose to limit the computation to the 10 closest cluster to reduce calculation time given that clusters that are far away are not expected to have high level of correlation and we only keep the highest value as parameter **b**.

In an ideal scenario where clusters are entirely independent of each other, the internal correlation approaches 1, and the external correlation approaches 0, resulting in a silhouette score close to 1. By examining the distribution of silhouette values for each threshold, we can select the threshold that yields the highest silhouette value.

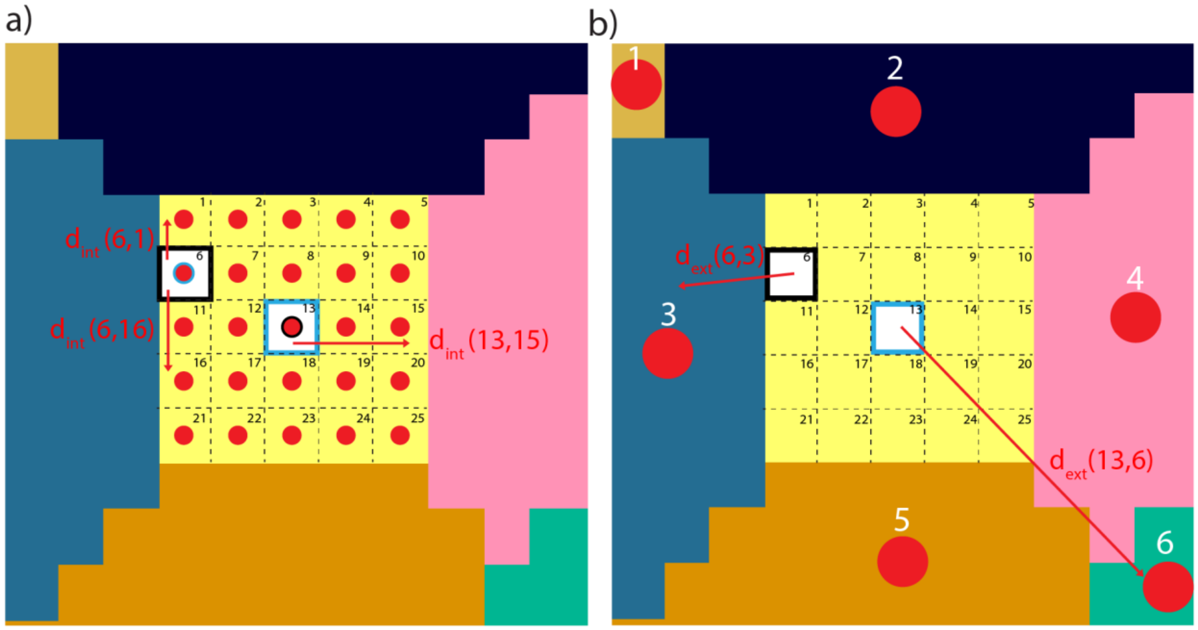


**Figure S3.1** Silhouette calculation schematic. The red disk show to what the two exemplary pixels are correlated to, in a) to pixel belonging to the same cluster, in b) to the time-dependent traces averaged over all pixel in the designated cluster. Examples of $d_{int}$ and $d_{ext}$ for the exemplary pixels are given on the figure. a) Schematic of internal correlation. For simplicity, only two pixels are represented, in white with a black border (close to cluster boundary) and in white with a blue border (pixel in the middle of the cluster). We look at the correlation of each pixel with the pixels belonging in the same cluster (here yellow cluster). The pixel present at the edge of the cluster (black border pixel) will typically have some amount of crosstalk (due to the diffraction limit) with the neighbouring cluster leading to slightly lower internal correlation than the blue-bordered pixel which is more at the centre. b) Schematic of external correlation. The correlation of each pixel with neighbouring cluster is calculated as represented by big red circles. The highest correlation is chosen as the external correlation to be compared with. Again, due to the crosstalk, the black-bordered pixel will have a higher external correlation than the blue-bordered pixel.

In this step, we introduce a modification by multiplying the silhouette by the proportion of the image that undergoes treatment (pixel clustered/total number of pixels). This adjustment penalizes the treatment of fewer pixels, that is, the use of larger thresholds. This is essential because, as the threshold value increases, fewer pixels exhibit the required level of correlation stipulated by the threshold. Consequently, only a small portion of the image undergoes processing, and in such cases, the resulting clustering may lack significance.

# Note SIV – Resolution of the method

Given that CLIM is an imaging technique designed to identify clusters of pixels with high correlation, determining the resolution limit of these clusters is crucial for understanding how it influences the analysis of photoluminescence (PL) fluctuation kinetics. The theoretical resolution of our optical system, with a numerical aperture (NA) of 0.6 and an emission wavelength of 780 nm, can be estimated using Abbé's criterion[2]:

$$Resolution = \frac{\lambda}{2\,NA} = \frac{780\,nm}{2 \cdot 0.6} = 650\,nm.$$

With a pixel size of 200 nm (our setup) the Nyquist criterion is respected. In CLIM, two types of resolution must be considered: the resolution of the **correlation map** and that of the **clustering**. These outputs need separate discussions, as the correlation map represents a continuous signal while the clustering results in a binarized mask.

## Resolution of the cluster map

The clustering algorithm in CLIM can potentially separate two regions with a greater precision than the diffraction limit. This is because, even if signals from the neighboring regions overlap, the distinct fluctuation patterns can still be distinguished, e.g., differentiating the fluctuation of particle A with some contribution from particle B, from the opposite case. Using the extra information from the correlation can lead to sub-resolution separation of two clusters.

To investigate this, we performed simulations (see Note SII for details) of two Gaussian emitters with distinct fluctuations. The Houston criterion[3] considers the diffraction limit to be the full width half maximum (FWHM) of the point spread function (PSF). Using this definition, we can easily test the ability of CLIM to separate two differently fluctuating emitters as a function of the distance between them. Since the data is simulated, the absolute distance units are not relevant, and instead, we express the distance relative to FWHM. Of note, in this case, any distance below unity indicates that the two emitters are closer than the diffraction limit according to the aforementioned Houston criterion.

In practice, we simulated emitters with varying distances, from 0.1 to 2 times the FWHM, and adjusted the number of pixels per FWHM to simulate different sampling rates (the number of pixels sampling the simulated PSF's FWHM). CLIM was then applied to assess the minimum separable distance between clusters.

The results (Fig. S4.1) show that the clustering method achieves a resolution approximately twice as good as the diffraction limit. Importantly, this enhanced resolution is unaffected by the sampling rate of the PSF's FWHM (Fig. S4.1c). However, it should be noted that when clusters are closer than the FWHM of the PSF, there will be signal overlapping/mixing, which needs to be considered when interpreting the PL kinetics data.

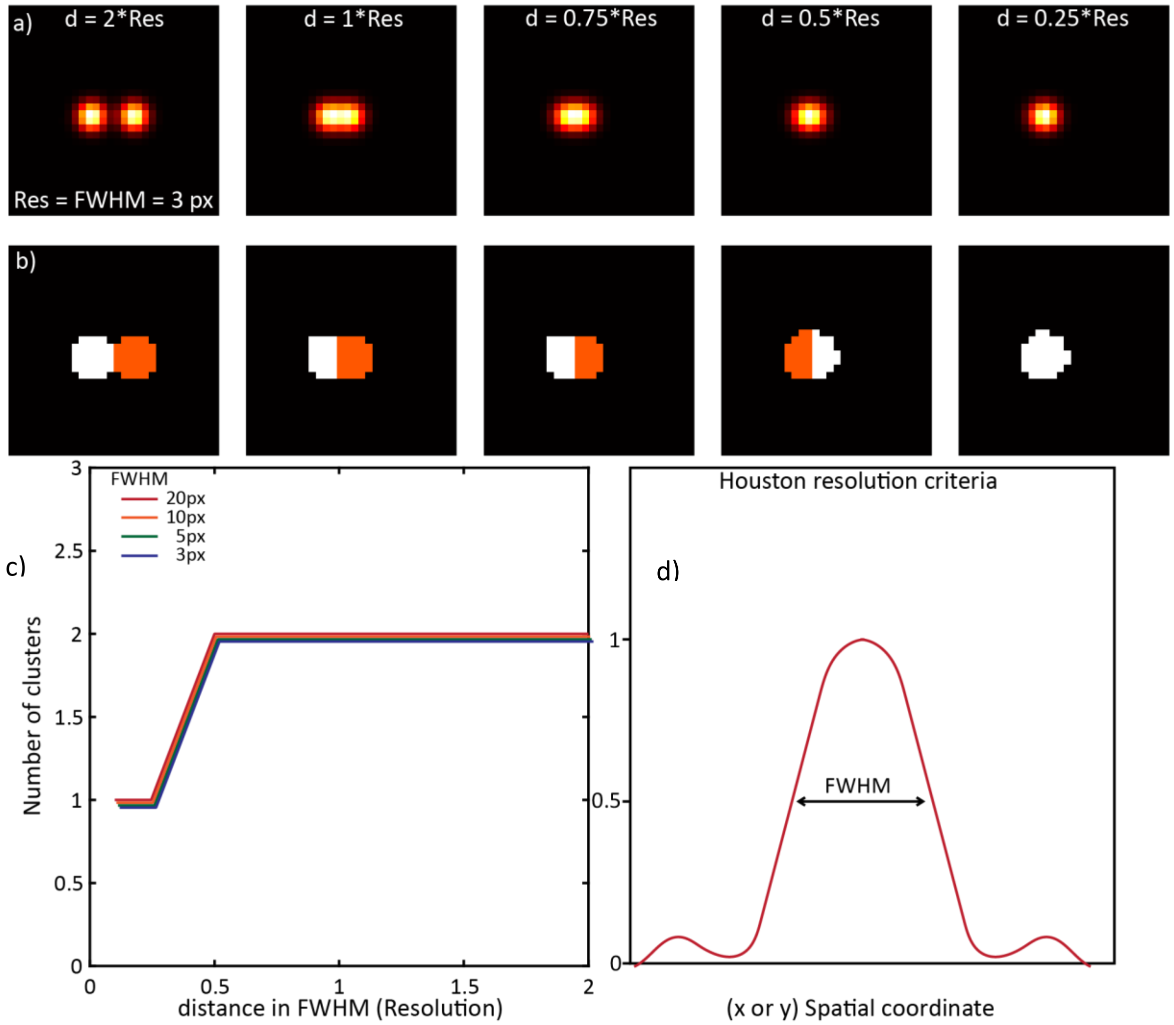


**Figure S4.1** Resolution limit of CLIM's cluster map. To gain insight on the resolution limit of the clustering, we simulated 2 emitters and varied the distance between them to see when the clustering would not be able to distinguish them. According to the Houston criterion, the FWHM of the PSF can be used to describe the resolution limit of a system. a) Images of two simulated point emitter for different distance between them varying between 0.25 and 2 times their simulated FWHM. b) Cluster map corresponding to the two emitters shown in a). c) Number of clusters found by CLIM as a function of the distance between the two emitters expressed in relative to the FWHM of the PSF. Two emitters can be distinguished as long as d>0.5*FWHM indicating the resolution to be around half the diffraction limit. d) Graphic representation of the Houston resolution criteria used here.

## Resolution of the correlation map

For the correlation map, the grains we observe are larger than the diffraction limit, consequently, we cannot use the same approach as in the previous section with the FWHM of the PSF. However, for these samples, CLIM's correlation map could display the grain boundaries as confirmed by SEM images. Therefore, since a boundary should ideally have close to zero width, we can treat the measured width of a boundary in CLIM's correlation image as a proxy for resolution.

To estimate the resolution, we analyze the profile of these grain boundaries. By examining the complementary of the correlation map (defined as 1−correlation map, see Fig. S4.2b), the boundary width is found to be approximately 2.5-3 pixels, which corresponds to 500-600 nm, just below the theoretical diffraction limit of our optical system. Considering the width of a boundary as resolution may seem counter intuitive compared to typical resolution determination which would use the grain instead. However, in this view, we can picture one grain between two boundaries: As the size of the grain reduces, the two boundaries get closer and closer to one another, until they can no longer be distinguished. The limit in this situation is how broad the boundary is on the image, and it will ultimately limit the smallest grain we can observe. Therefore, given that the grains are above the resolution it appears more appropriate to use this definition, which is the minimal distance between two boundaries to be distinguishable.

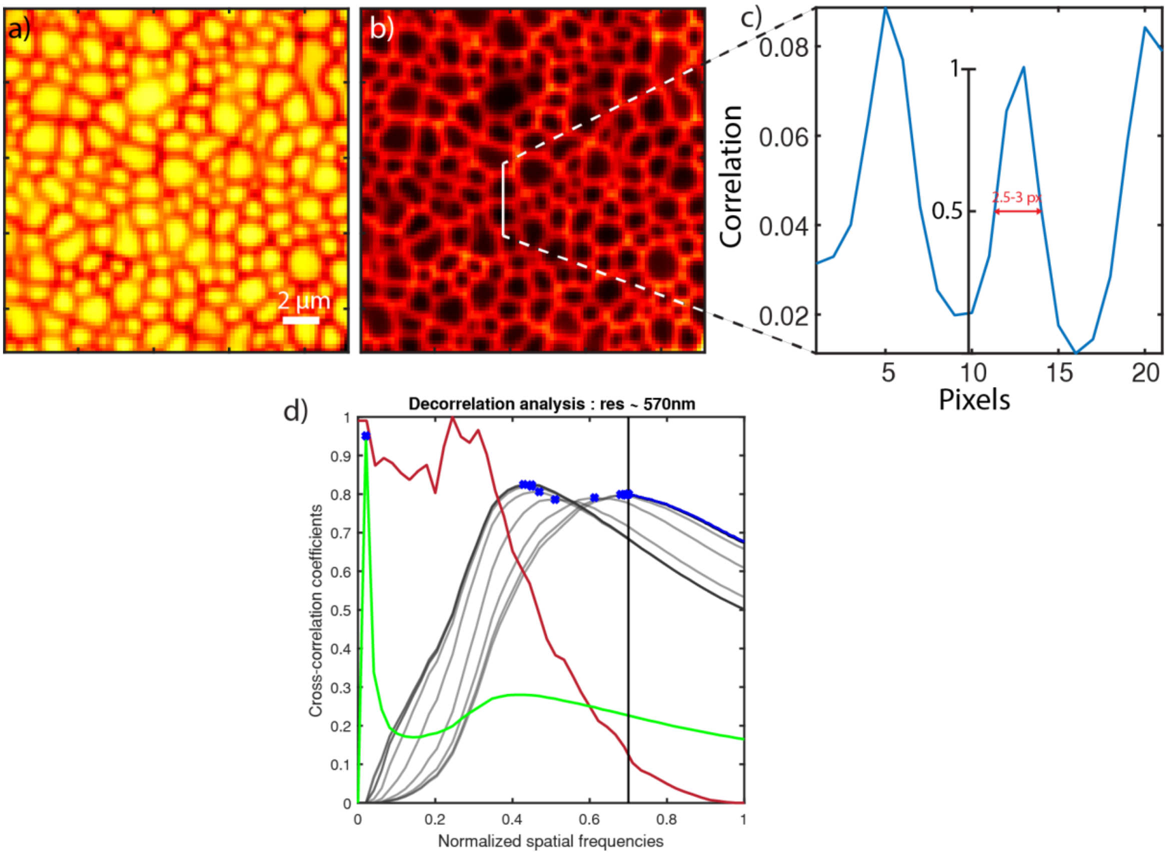


**Figure S4.2** Resolution of CLIM's correlation map. a) CLIM correlation map of MAPI-LG b) Complementary correlation map of MAPI-LG (1-correlation map) showing a line from which an intensity profile was extracted. c) Resulting line profile showing multiple grain boundaries, the central one was characterized to be 2.5-3 pixels which is 500-600 nm, very close to the expected diffraction limit of the system. d) Results of decorrelation analysis (Descloux et al. Nat. Methods, 2019) of the image shown in b). This method yields a resolution of 570nm, close to the theoretical diffraction limit calculated by Abbé equation.

As an alternative method, we applied on the same image (Figure S4.2b), the decorrelation analysis developed by Descloux et al.[2] which provides a parameter-free estimation of resolution based on image spatial decorrelation. This method essentially determined the finest details observable in the image. This method estimated the resolution at 570 nm, slightly (~15%) better than the diffraction-limited resolution and similar to the line profile approach. Although the correlation map doesn't drastically improve the theoretical resolution, it reveals grain boundaries with remarkable clarity, which standard fluorescence microscopy fails to achieve.

## Note SV – Influence of uncorrelated and correlated signals

In CLIM, there are two types of parasitic signals that can affect the clustering outcome. 1) Uncorrelated signals, such as background noise. 2) Correlated signals such as photobleaching and enhancement. In both cases, we ran simulation in which 4 Gaussian emitters are placed close to each other such that their signals are mixed. We can then increase the amount of noise by adding Gaussian noise or by increasing/decreasing the base intensity level (see Note SI) of each emitter in the course of the simulation.

Uncorrelated signals

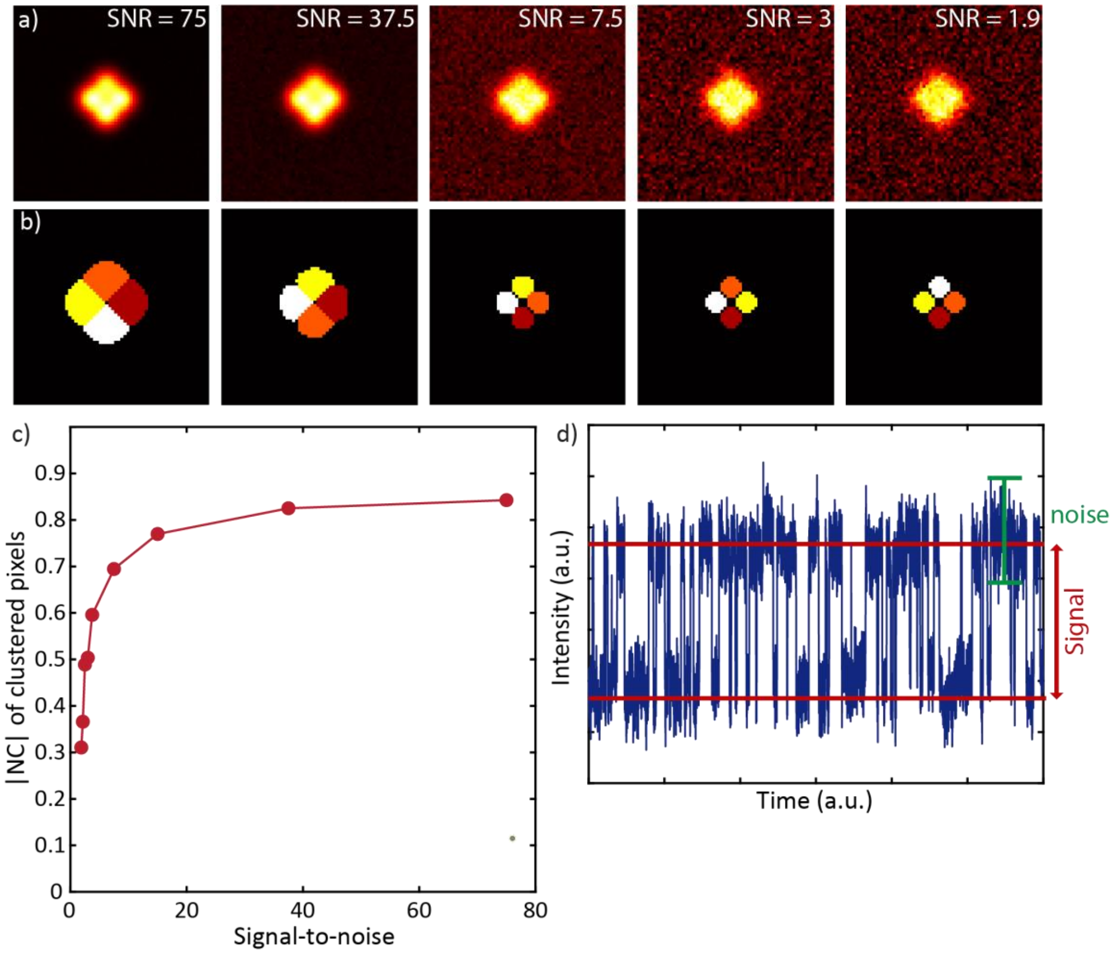


**Figure S5.1.** Influence of signal-to-noise on CLIM. a) Images of simulated movie at different signal-to-noise ratios b) corresponding CLIM cluster maps. c) Average correlation to neighbouring pixels (|NC|) of clustered pixel as a function of the signal-to-noise ratio. d). Exemplary simulated trace extracted using CLIM. To calculate the signal-to-noise ratio, the noise was defined as the standard deviation of the Gaussian noise added ranging from 10 to 400 counts, covering a range of SNR from 75 to 1.9 considering a signal of 750 counts. The signal was defined as the amplitude of the blinking which was calculated using the difference between the bottom and top 10th percentile. Even at the lowest SNR, the clustering algorithm could still correctly assign the 4 clusters.

To compare different conditions of uncorrelated noise, we can easily keep the blinking parameter (amplitude =750 counts, switching frequency 5%) constant and change the amount of gaussian noise that we add. Then, we can observe how the signal-to-noise impacts the CLIM outputs. Given that, in the data we treat, the main source of correlation would be intensity fluctuations, it is relevant to use

the amplitude of these fluctuations (blinking amplitude) as the signal which we then divide by the standard deviation of the noise added. Figure S5.1d shows an exemplary trace with the signal and the noise depicted. Gaussian noise added ranged from 10 to 400 counts, covering a range of SNR from 75 to 1.9 considering a fluctuation amplitude (signal) of 750 counts.

Fig. S5.1 shows the results for different signal to noise ratios. We can see that the algorithm can detect the cluster even for signal to noise ratios lower than 2. This is likely because random noise only lowers the correlation (as can be seen from the lowering average correlation to neighbors |NC| from the clustered pixels in panel b)) but doesn't mask the underlying correlated signal. This is valid as long as the standard deviation of the random fluctuation does not surpass the individual fluctuations of the sample.

Correlated signals

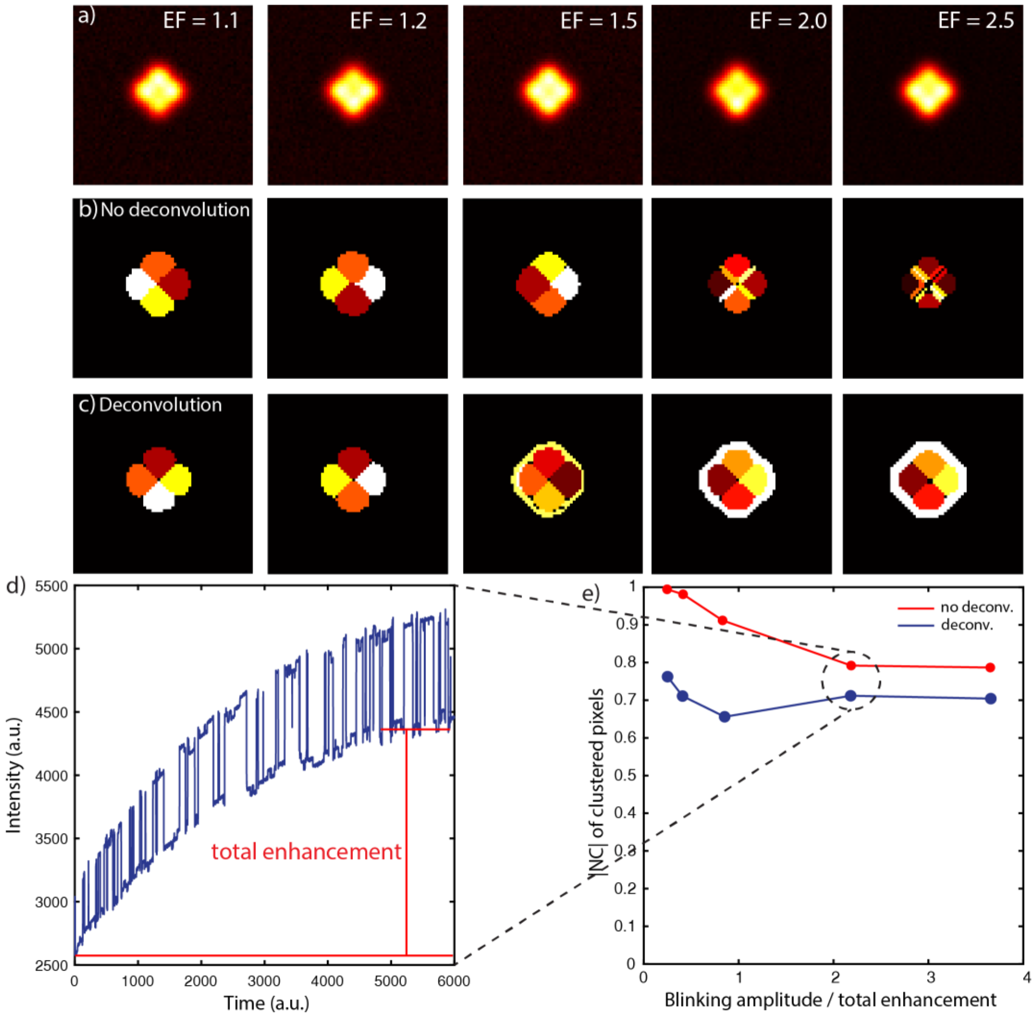


**Figure S5.2.** Impact of correlated signal (here, enhancement) on CLIM. a) Images of a simulated system of 4 blinking emitters for different enhancement factors (EF), ranging from 1.1 to 2.5. b) Resulting cluster maps without applying the deconvolution showing artefact in the cluster map at high enhancement factors. c) Resulting cluster maps with deconvolution showing the 4 regions correctly separated, albeit with some minor artefacts at the interface between the signal and the background. d) example of simulated intensity trace with simultaneous enhancement and blinking for EF = 2. e) Average correlation as a function of the ratio of blinking amplitude to enhancement, for deconvoluted and non-deconvoluted data. The deconvolution minimizes the effect of PL enhancement on clustering.

Examples of correlated signal include continuous change in intensity such as enhancement or bleaching, laser induced fluctuations or sample vibration. Although investigating all potential sources of correlated signals exceeds the scope of this study, it is worthwhile to explore gradual intensity changes which are commonly encountered in imaging of semiconductor materials as well as dye-labelled structures.

To explore this, we also simulated four nearby blinking emitters and gradually increased their base intensity during the course of the simulation. The total enhancement factor was defined as the difference between the base intensity in the last simulated frame compared to the first one (see Figure S5.2d. The enhancement factor (EF) is the ratio between the final base intensity to the initial one and was ranging from 1.1 to 2.5 times. The blinking amplitude was defined as the difference between the base intensity ("ON" state) and the quenched intensity ("OFF" state) and was simulated to ensure that the relative blinking amplitude was constant.

To compare various conditions, we computed the ratio between the blinking amplitude and the total absolute intensity enhancement. Fig. S5.2 displays the simulation results and their impact on CLIM outputs.

Fig. S5.2a-c shows that, at low enhancement factors, the cluster map remains unaffected by the enhancement. However, when the enhancement surpasses the blinking amplitude, artifacts start to appear in the CLIM clusters. Using deconvolution of the average intensity (see Note SIII) effectively reduces these artifacts to the signal/background interface and maintains emitter separation.

Figure S5.2.e shows the |NC| of clustered pixels as a function of the blinking amplitude to total enhancement ratio (blinking amplitude of 750, with total enhancement of 1500 would yield a ratio of 0.5). We observed that the average correlation increases significantly when the total enhancement increases (ratio decreases), the effect is almost totally suppressed with the deconvolution except for the total enhancement values that are higher than the blinking amplitude.

# Note SVI – CLIM on $MAPbI_3$ films with different grain sizes

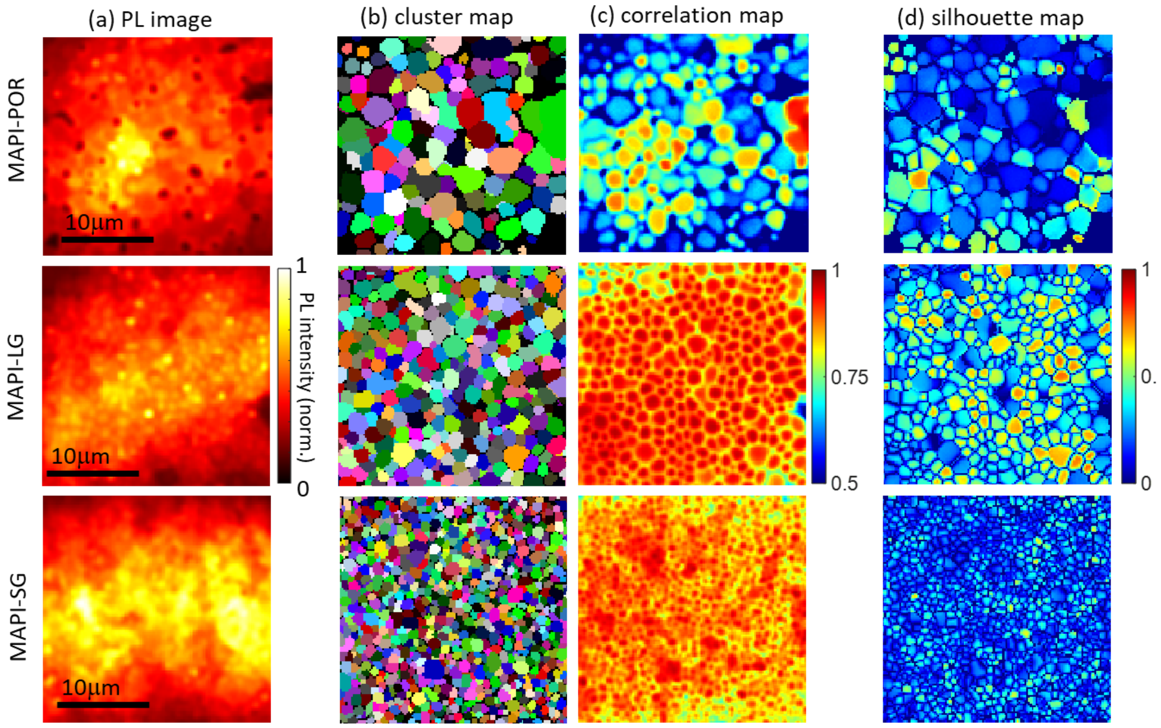


**Figure S6.1.** CLIM results on three $MAPbI_3$ films with different grain sizes. a) PL image, b) cluster map, c) correlation map and d) silhouette map of porous (MAPI-POR), large grain (MAPI-LG), and the small grain (MAPI-SG) films.

## Note SVII – Overlay of CLIM outputs.

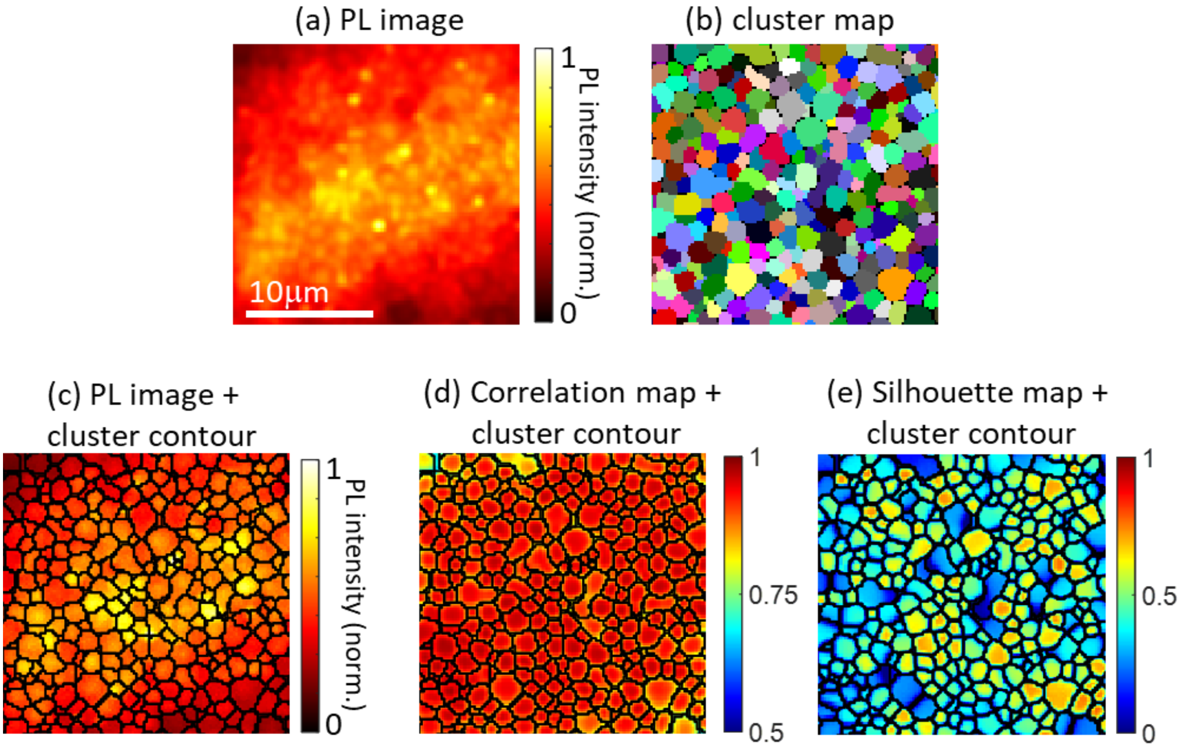


**Figure S7.1.** Overlay of different CLIM outputs, a) PL image and b) cluster map of the same area of a MAPI-LG film. Overlay of c) the PL image and the contour of the cluster map, d) the correlation map and contour of the cluster map and e) the silhouette map and contour of the cluster map.

## Note SVIII – Analysis of PL fluctuation kinetics

In typical fluorescence blinking studies, samples consist of individual, well-separated objects that can be resolved in fluorescence microscopy images. The intensity of each individual object is measured by integrating over all pixels (when using a CCD detection) belonging to the whole time series of the object's image (a movie). The resulting time-dependent signal is referred to as a fluorescence intensity trace. However, this conventional approach cannot be directly applied to continuous fluorescent films. CLIM offers a solution to this problem by segmenting the PL image into regions of high correlation (clusters). This segmentation allows the extraction of PL intensity traces from individual clusters by integrating the intensity over these regions and monitoring them over time. Once these intensity traces are obtained, they can be further analyzed. In this study, we can confidently confirm that each cluster corresponds to an individual grain, which validates the methodology.

Traditionally, the analysis of PL fluctuation kinetics relies on the probability distribution of ON and OFF events within the PL traces. These ON and OFF events are determined by applying a threshold, which can introduce statistical bias into the results unless the traces exhibit only two distinctly defined intensity levels.[4]

Recently some of us have demonstrated power spectral density (PSD) estimation as a potential alternative for the analysis of fluctuating PL traces in MHPs.[5] Fluctuations in MHPs arise due to the metastability of nonradiative recombination centers within the materials.

Power spectral density estimation of photoluminescence fluctuations.

### Theory

Power spectral density (PSD) is a widely used statistical analysis method for fluctuating signal (I(t)). It finds increased utility in studying PL fluctuations of semiconductor materials due to its unbiased approach in contrast to the commonly used analysis of ON and OFF times.[4] The PSD can be calculated from the intensity autocorrelation function. Considering X(t) = I(t) − ⟨I(t)⟩ as the time-dependent deviation of the signal from its time-averaged value, the autocorrelation function can be written as:

$$K(t) = \langle x(t+t')x(t)\rangle \tag{S8.1}$$

The PSD is calculated through the cosine transformation of the autocorrelation function according to the Wiener-Khinchin theorem.

$$PSD(f) = \int_{-\infty}^{\infty} K(t)\cos(2\pi f t)dt \tag{S8.2}$$

Where $f$ is the frequency.

PSD is proportional to the square of the mean signal and square of the relative fluctuation amplitude $\Delta I/Imax$. Details of the theory and algorithm of the power spectral density estimation are provided in previously published work.[5–7]

### Modelling of PSD

In emitting semiconductor systems, PL fluctuation results from the combined response of several types of nonradiative recombination processes. Each of these nonradiative channels introduces a distinct two-level system (TLS) in the framework of multiple recombination center (MRC) model.[8] Depending on whether the contribution arises from one or several types of TLSs with significantly different switching rates, the PSD can exhibit a range of signatures, spanning from a pure Lorentzian to a stretched Lorentzian to a pure power-law behavior, each with variable slopes:

$$PSD(f) = \frac{A}{1 + (f/f_0)^{\beta}} \qquad \text{(S8.3)}$$

$$PSD(f) = \frac{A'}{f^{\beta}} \qquad \text{(S8.4)}$$

Here $f_0$ = 1/(2πτ) and τ is the characteristic fluctuation time. β is the stretching exponent. β = 2 for fluctuations caused by one or several TLSs with the same characteristic frequency.

In the case of PL fluctuations in sub-micrometer MAPI crystals, ranging in size from 100 to 500 nanometers, we observed PSD profiles characterized by saturation at low frequencies and a power-law dependence at high frequencies, featuring a slope (β) in the range from 1.6 to 2. Remarkably, certain crystals exhibited PSDs very close to a Lorentzian shape (β = 2), indicating the presence of TLSs with well-defined characteristic timescales within the range of 0.5 to 10 seconds.[5]

CLIM revealed that the PL of individual MHP grains in the film fluctuates majorly independently under ambient conditions, indicating the presence of several efficient metastable NR recombination centers, often referred to as "super-traps".[9] Furthermore, the extracted intensity traces do not exhibit well-defined intensity levels as expected for a single metastable super-trap (a TLS), raising questions about the number and properties of these metastable super-traps within each grain.

To address these questions, simulations were conducted by assuming a combination of multiple NR recombination centers fluctuating as TLSs using the Stern-Volmer equation for quantum yield [10] (see Note SIX). The simulations shown in Fig. S9.1 revealed that more than 30 metastable NR recombination centers are needed to qualitatively mimic the time-dependent behavior of PL in individual grains within the film. However, this is a rough estimation that depends on the ON and OFF time of each TLS as well as their quenching efficiency.

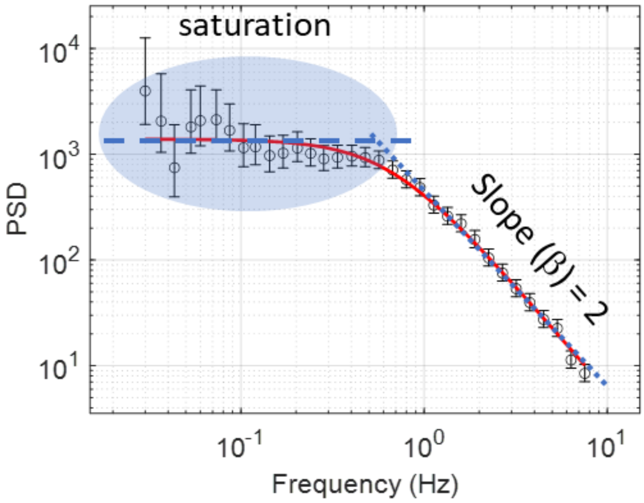


**Figure S8.1.** PSD derived from an intensity trace of a cluster of MAPI-LG film. Low-frequency region (blue-shaded area, dashed line) shows saturation suggesting a characteristic timescale of PL fluctuation. The slope (β, blue dotted line) is 2, suggesting Lorentzian behavior.

As depicted in Fig. S8.1, PSDs of each cluster obtained from CLIM demonstrate stretched Lorentzian behavior with β very close to 2 (1.8-2.1) suggesting that fluctuations in each grain are dominated by

only a single characteristic time. This overall behavior was observed consistently in all studied samples (MAPI-LG, MAPI-POR and MAPI-SG), with some differences. Generally, smaller grain size led to a decrease of β, deviating further from the value of 2 (indicative of a single characteristic time) towards lower values (1.8 – 1.7) as shown in Fig. 4b of the main text. This suggests that additional fluctuation processes at faster time scales become important in smaller grains, consistent with the idea that smaller grains exhibit higher NR processes compared to larger grains.[11] This is also the case when analyzing the size dependence within the same sample (see Note SX).

Many PSDs exhibit deviations from the constant level (saturation) in the low-frequency range, indicating the presence of slower processes compared to the characteristic fluctuation time obtained with the stretched Lorentzian function. This is consistent with previous results obtained on individual MAPI crystals.[3]

## PSD mapping of the clusters

Once we obtained the values of β and τ for each of the individual grains, we can map these values back on the cluster map where the colors represent either the β or the τ values. Fig. S8.2 shows how such mapping looks for the MAPI-LG sample. This mapping can be extremely useful to gain insight into the defects present in the materials and their spatial distribution.

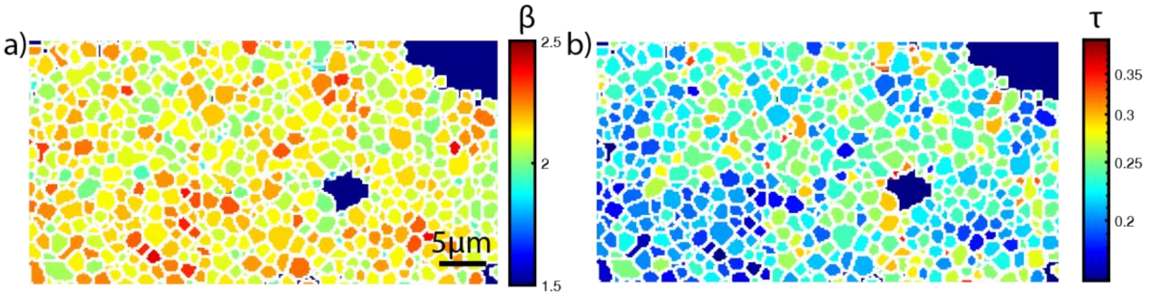


**Figure S8.2.** PSD mapping of the clusters. a) Mapping of β values corresponding to each cluster in the MAPI-LG film shows some heterogeneity but most of the values are close to 2 or slightly above. b) Mapping of the τ shows some heterogeneity but mostly ranges from 0.2-0.35 s. Mapping of β and τ provides an overview of the defect types and their distribution across the film.

## Note SIX – Blinking simulations

To simulate blinking we use the Stern-Volmer equation for the quantum yield Φ as given below.[10]

$$\Phi = \frac{1}{1 + \sum_i s_i \epsilon_i} \tag{S9.1}$$

Where:

- $s_i$ represents the state of metastable NR recombination center i, taking a value of 1 if the center is active (quenches PL) and 0 if inactive.
- $\epsilon_i$ is the efficiency coefficient of the metastable NR recombination center i. A higher value of $\epsilon_i$ corresponds to a lower quantum yield due to increased quenching when the center becomes active.

For simplicity and based on the observed PL kinetics with β≈2 in the PSDs of the traces, we assume a specific type of trap. Consequently, the efficiency of all NR centers and their switching probabilities are considered equal. Thus, the equation can be simplified as follows:

$$\Phi = \frac{1}{1 + n'\epsilon} \tag{S9.2}$$

Here, n' is the number of traps in the active state, and ϵ denotes the quenching efficiency of the trap.

For the simulation, we introduced a certain number of traps, each with a specific efficiency and a probability of being active or inactive. To determine their states at each time point, we employed a random number generator (in MATLAB), ensuring an accurate representation of the traps' probabilities of being active or inactive. Once the states of all traps were determined for a specific time point, we calculated the intensity at that moment using (Eq. S9.2). To incorporate the exposure time into the simulation, we generated the trace with a time resolution 10x higher than the targeted one and subsequently downsized it by a factor of 10 to match the resolution of the experimental data.

Figure S8 shows exemplary simulated traces for various number of traps. We can see that the relative amplitude of the fluctuations decreases with increasing number of traps, due to ensemble averaging. The simulation parameters were chosen to mimic as much as possible the experimental behavior. Hence, the quenching efficiency at 20% assuming that it could not be too high since the PL "OFF" state is quite far from the background level in the experimental data. The probability to switch from active to inactive state for the trap were chosen to be both equal to 5% because it yielded a switching rate in the power spectra density that was close to the experiment (~1sec).

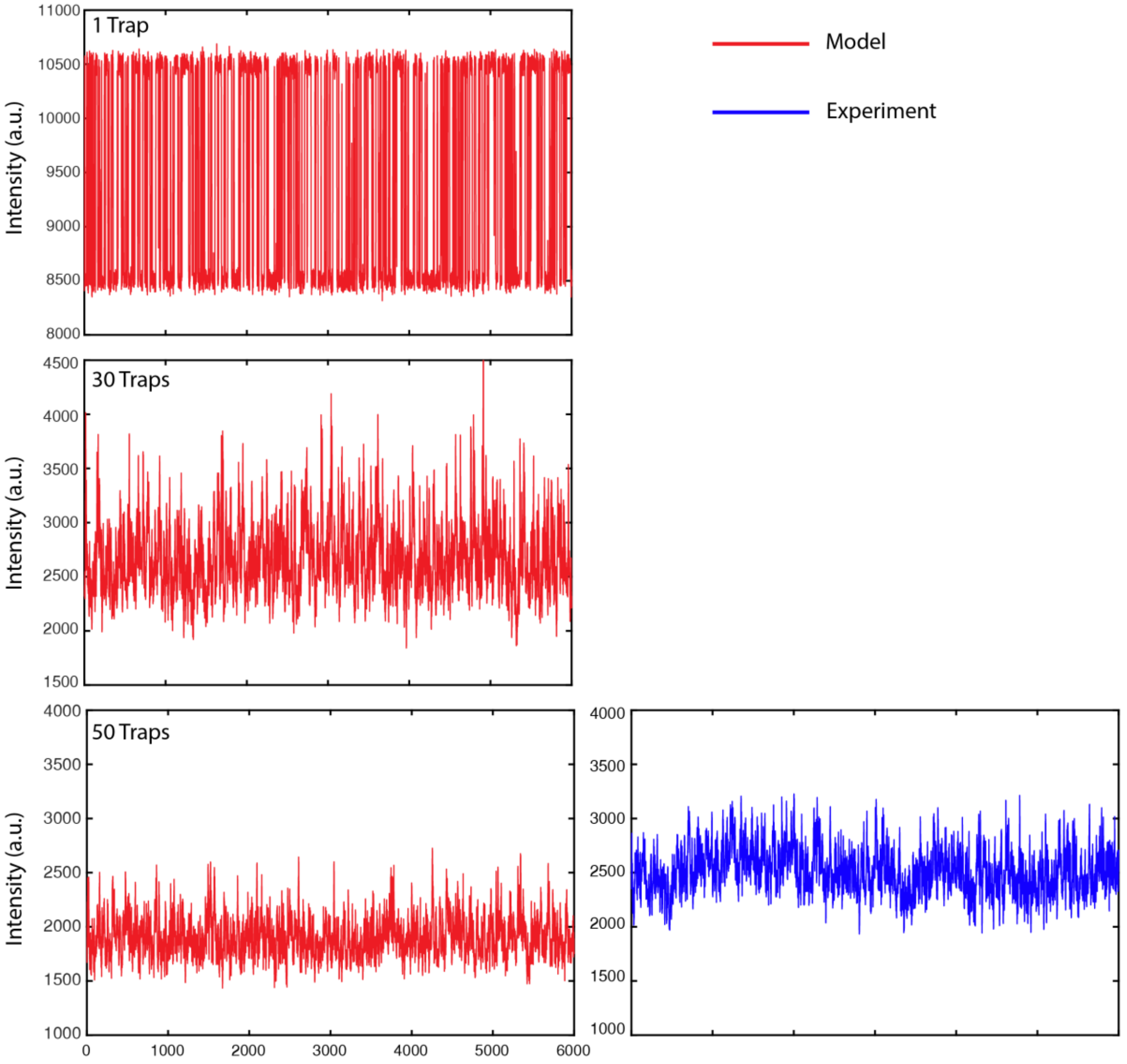


**Figure S9.1.** Blinking model vs experimental data. The red curves show our simple blinking simulation for 1, 30 and 50 traps using 20% quenching efficiency per trap and a 5% chance to switch from active to inactive and vice versa. The blue curves show an exemplary experimental data. It is quite clear that a fairly large number of traps need (30 to 50) to be present in the system to mimic the experimental data with the current simulation settings.

Note SX – Size dependence of the β and τ values obtained from the stretched Lorentzian fitting of the PSDs of the traces from the same sample.

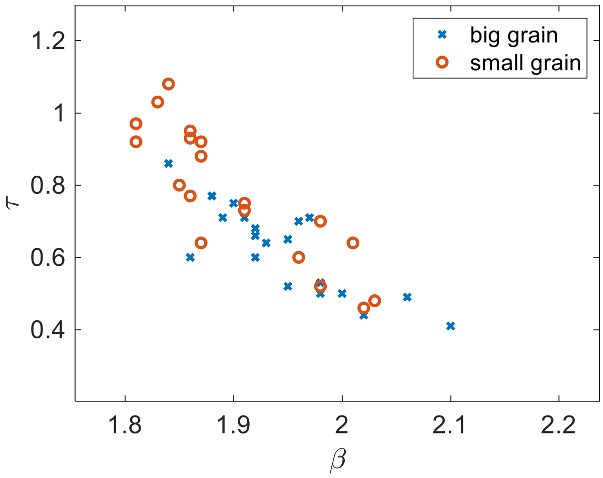


**Figure S10.1.** Size dependence of the β and τ values obtained from the stretched Lorentzian fitting of the PSDs of the traces from the same sample (MAPI-LG). Within the large grain sample the size of grains varies and there are comparatively small and big grains present. This comparison suggests that smaller grain tend to have smaller beta values, similar to what is observed across the samples with different average grain size (MAPI-LG, MAPI-SG).

# Note SXI – Device structure, performances and CLIM imaging

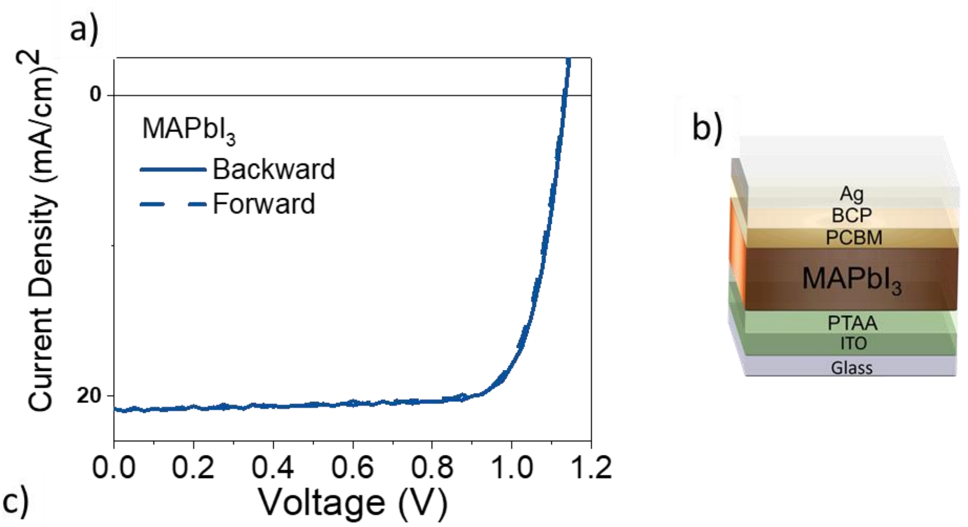


c)

| | Jsc (mA/cm²) | Voc (V) | FF (%) | PCE (%) |
|---|---|---|---|---|
| Forward | 20.85 | 1.13 | 78.12 | 18.42 |
| Backward | 20.85 | 1.13 | 78.29 | 18.52 |

**Figure S11.1.** Device structure and performance. a) I-V curve of the measured perovskite based solar cell. b) Schematic of the solar cell device structure. c) Parameters of the solar cell.

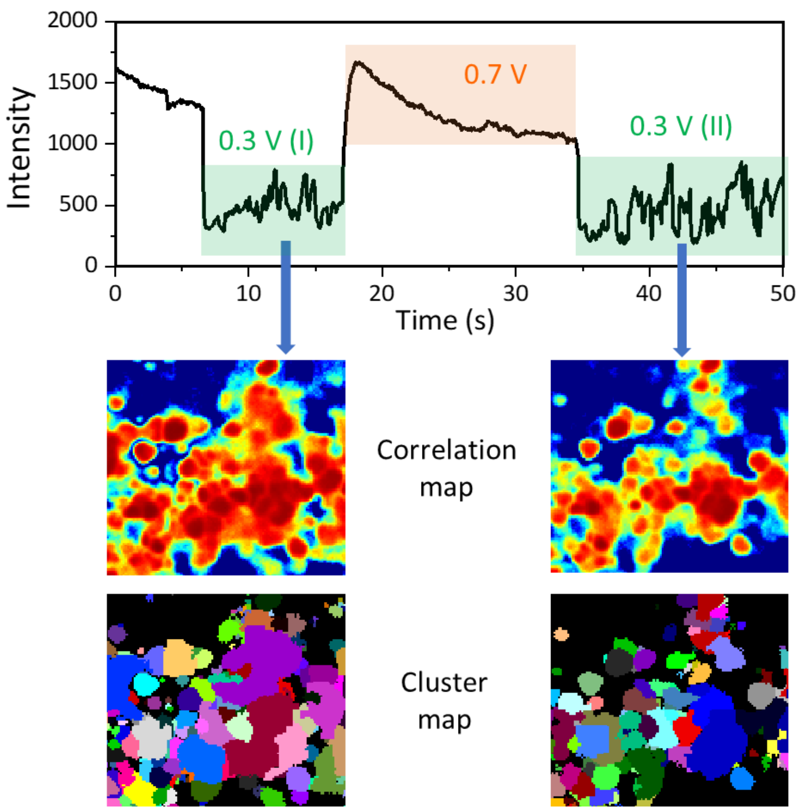


**Figure S11.2.** CLIM imaging of MAPI solar cells at two instances of 0.3 V operating conditions. These two instances are separated by a time interval of 17 seconds when 0.7 V condition was applied.

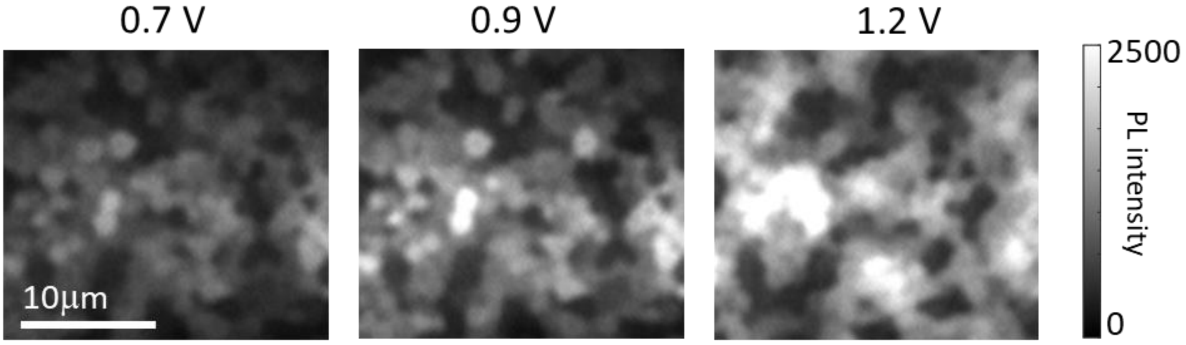


**Figure S11.3.** Photoluminescence intensity images of the MAPI solar cell at three different operating conditions as represented by the corresponding voltages.

# Note SXII – Characteristics of PL Intensity jumps

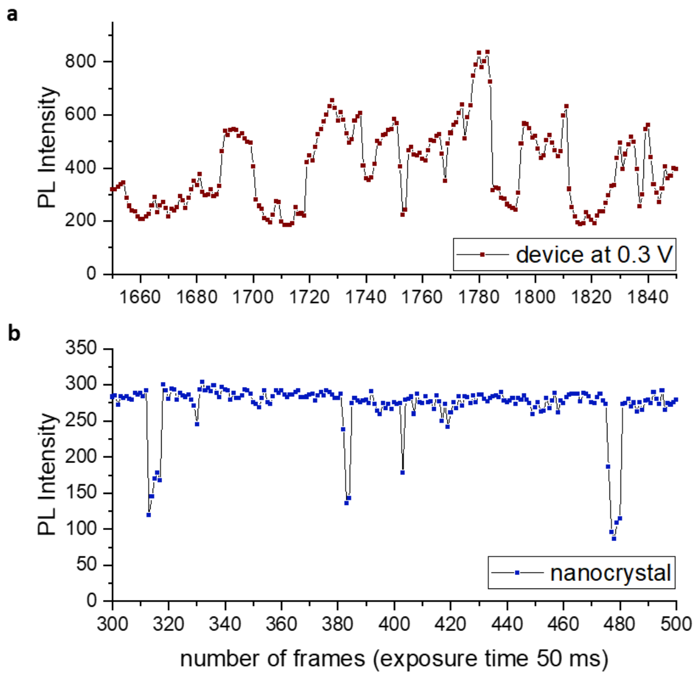


**Figure S12.1** Photoluminescence intensity traces corresponding to (a) a CLIM cluster in the solar cell operating at 0.3 V under 485 nm excitation with a power density of 0.15 W/cm$^2$. (b) Photoluminescence intensity traces of a 100 nm crystal on a coverslip at the similar microscopic experimental conditions. Here each frame has 50 millisecond exposure time. Each data point represents intensity at each frame of the movie.

# References


[1] P. J. Rousseeuw, *J. Comput. Appl. Math.* **1987**, *20*, 53.
[2] E. Abbe, *Arch. Für Mikrosk. Anat.* **1873**, *9*, 413.
[3] W. V. Houston, *Phys. Rev.* **1927**, *29*, 478.
[4] C. H. Crouch, O. Sauter, X. Wu, R. Purcell, C. Querner, M. Drndic, M. Pelton, *Nano Lett.* **2010**, *10*, 1692.
[5] S. Seth, E. A. Podshivaylov, J. Li, M. Gerhard, A. Kiligaridis, P. A. Frantsuzov, I. G. Scheblykin, *Adv. Energy Mater.* **2021**, *11*, 2102449.
[6] E. A. Podshivaylov, P. A. Frantsuzov, **2023**, DOI https://doi.org/10.5281/zenodo.8027381.
[7] E. A. Podshivaylov, M. A. Kniazeva, A. O. Tarasevich, I. Yu. Eremchev, A. V. Naumov, P. A. Frantsuzov, *J. Mater. Chem. C* **2023**, 8570.
[8] P. A. Frantsuzov, S. Volkan-Kacso, B. Janko, *Phys. Rev. Lett.* **2009**, *103*, 1.
[9] A. Merdasa, Y. Tian, R. Camacho, A. Dobrovolsky, E. Debroye, E. L. Unger, J. Hofkens, V. Sundström, I. G. Scheblykin, *ACS Nano* **2017**, *11*, 5391.
[10] I. G. Scheblykin, *Adv. Energy Mater.* **2020**, *10*, 2001724.
[11] Q. An, F. Paulus, D. Becker-Koch, C. Cho, Q. Sun, A. Weu, S. Bitton, N. Tessler, Y. Vaynzof, *Matter* **2021**, *4*, 1683.
